# A Cognitive Biotype of Depression and Symptoms, Behavior Measures, Neural Circuits, and Differential Treatment Outcomes: A Prespecified Secondary Analysis of a Randomized Clinical Trial

**DOI:** 10.1001/jamanetworkopen.2023.18411

**Published:** 2023-06-15

**Authors:** Laura M. Hack, Leonardo Tozzi, Samantha Zenteno, Alisa M. Olmsted, Rachel Hilton, Jenna Jubeir, Mayuresh S. Korgaonkar, Alan F. Schatzberg, Jerome A. Yesavage, Ruth O’Hara, Leanne M. Williams

**Affiliations:** 1 Department of Psychiatry and Behavioral Sciences, Stanford University, Stanford, California; 2 Sierra-Pacific Mental Illness Research, Education, and Clinical Center (MIRECC) Veterans Affairs Palo Alto Health Care System, Palo Alto, California; 3 Brain Dynamic Centre, Westmead Institute for Medical Research, The University of Sydney, Westmead, New South Wales, Australia

## Abstract

**Importance:**

Cognitive deficits in depression have been associated with poor functional capacity, frontal neural circuit dysfunction, and worse response to conventional antidepressants. However, it is not known whether these impairments combine together to identify a specific cognitive subgroup (or “biotype”) of individuals with major depressive disorder (MDD), and the extent to which these impairments mediate antidepressant outcomes.

**Objective:**

To undertake a systematic test of the validity of a proposed cognitive biotype of MDD across neural circuit, symptom, social occupational function, and treatment outcome modalities.

**Design, Setting, and Participants:**

This secondary analysis of a randomized clinical trial implemented data-driven clustering in findings from the International Study to Predict Optimized Treatment in Depression, a pragmatic biomarker trial in which patients with MDD were randomized in a 1:1:1 ratio to antidepressant treatment with escitalopram, sertraline, or venlafaxine extended-release and assessed at baseline and 8 weeks on multimodal outcomes between December 1, 2008, and September 30, 2013. Eligible patients were medication-free outpatients with nonpsychotic MDD in at least the moderate range, and were recruited from 17 clinical and academic practices; a subset of these patients underwent functional magnetic resonance imaging. This prespecified secondary analysis was performed between June 10, 2022, and April 21, 2023.

**Main Outcomes and Measures:**

Pretreatment and posttreatment behavioral measures of cognitive performance across 9 domains, depression symptoms assessed using 2 standard depression scales, and psychosocial function assessed using the Social and Occupational Functioning Assessment Scale and World Health Organization Quality of Life scale were analyzed. Neural circuit function engaged during a cognitive control task was measured using functional magnetic resonance imaging.

**Results:**

A total of 1008 patients (571 [56.6%] female; mean [SD] age, 37.8 [12.6] years) participated in the overall trial and 96 patients participated in the imaging substudy (45 [46.7%] female; mean [SD] age, 34.5 [13.5] years). Cluster analysis identified what may be referred to as a cognitive biotype of 27% of depressed patients with prominent behavioral impairment in executive function and response inhibition domains of cognitive control. This biotype was characterized by a specific profile of pretreatment depressive symptoms, worse psychosocial functioning (*d* = −0.25; 95% CI, −0.39 to −0.11; *P* < .001), and reduced activation of the cognitive control circuit (right dorsolateral prefrontal cortex: *d* = −0.78; 95% CI, −1.28 to −0.27; *P* = *.*003). Remission was comparatively lower in the cognitive biotype positive subgroup (73 of 188 [38.8%] vs 250 of 524 [47.7%]; *P* = .04) and cognitive impairments persisted regardless of symptom change (executive function: *η_p_^2^* = 0.241; *P* *<* .001; response inhibition: *η_p_^2^* = 0.750; *P* < .001). The extent of symptom and functional change was specifically mediated by change in cognition but not the reverse.

**Conclusions and Relevance:**

Our findings suggest the presence of a cognitive biotype of depression with distinct neural correlates, and a functional clinical profile that responds poorly to standard antidepressants and instead may benefit from therapies specifically targeting cognitive dysfunction.

**Trial Registration:**

ClinicalTrials.gov Identifier: NCT00693849

## Key Points

**Question:**

How are objective cognitive deficits in depression associated with symptoms, neural circuits, and treatment outcomes?

**Findings:**

In this secondary analysis of a randomized clinical trial in 1008 patients with major depression, 27% exhibited pretreatment global cognitive impairment and significantly decreased brain response to a cognitive task as well as worse response to standard pharmacotherapy, defining what may be categorized as a cognitive biotype. Changes in cognitive symptoms over the course of treatment mediated the association between pretreatment cognitive status and improvement in overall symptoms and psychosocial functioning.

**Meaning:**

These results suggest that consideration of treatments targeting cognitive dysfunction in a subset of patients with depression is warranted to attain symptomatic and psychosocial improvement.

## Teaser

This secondary analysis of a randomized clinical trial tests whether a cognitive biotype is associated with diagnosed depression with symptoms, neural circuits, and treatment outcomes.

## Introduction

Major depressive disorder (MDD) is a serious, often chronic condition with a staggering burden that disrupts psychosocial, interpersonal, and workplace function. Cognitive impairment is recognized as a major contributor to both poor functional outcomes and lack of symptom relief from antidepressant treatments.^1^ Such impairment encompasses deficits in executive control, attention, and working memory relevant to the research domain criteria cognitive system^2^ and to the MDD *Diagnostic and Statistical Manual of Mental Disorders* (Fifth Edition, text revision) diagnostic criterion of poor concentration and indecisiveness.^3^ We undertook a systematic evaluation of what we describe as a cognitive dyscontrol biotype of depression, using behavioral measures to identify the biotype prevalence, clinical ratings to assess its association with symptoms and function, functional neuroimaging to reveal neural mechanistic underpinnings, and a randomized clinical trial to determine whether cognitive impairment is a causal mediator of poor outcomes following antidepressant treatment (ADT).

A growing pool of studies document cognitive impairment in MDD across the adult lifespan and assess treatments for it.^4,5,6^ These studies lack a consistent battery of cognitive measures and do not stratify depressed patients based on the extent of cognitive impairment, possibly leading to mixed treatment results on efficacy. Given this, we lack data specific to the patients with MDD distinguished by moderate-to-severe cognitive impairment, which may form a specific biotype subgroup.^1,7,8^

Our previous work has shown that patients with MDD, in young to mid-adulthood, are distinguished from healthy patients by a group mean reduction in cognitive performance, which persists despite ADTs^9^ and is implicated in nonremission.^10^ We have also observed that nonremission is characterized by a mean reduction in activation of the dorsolateral prefrontal cortex (dLPFC),^11^ a brain region essential in the cognitive control circuit, and by alterations in the connectivity of this circuit.^12^ In this study, we pursued a systematic, multimodal characterization of our prior proposed putative clinical biotype of depression called the cognitive dyscontrol biotype^13^ (hereafter referred to as *cognitive biotype* for brevity).

We used a machine learning method of cluster analysis to identify the cognitive biotype within the broader MDD diagnosis. Our primary research goal was to validate the cognitive biotype of depression by assessing whether it is distinguished by baseline symptom and function profiles, by baseline neural dysfunctions in the cognitive control circuit, and by poorer response to standard antidepressants. We also sought to determine whether lack of amelioration of cognitive impairment mediates poorer symptom and function outcomes during the treatment period (but not the reverse).

## Methods

### Overview and Patients

Data were from 1008 adults with MDD who participated in the International Study to Predict Optimized Treatment in Depression (iSPOT-D)^14^ and were enrolled between December 1, 2008, and September 30, 2013 (Supplement 1). Of these, 96 completed the iSPOT-D imaging substudy and were used in this analysis.^15^ The study protocol was reviewed and received institutional review board approval at each clinical site. Participants provided written informed consent. This prespecified secondary analysis was performed between June 10, 2022, and April 21, 2023. Both sample size and power calculations were established in the full sample.^14,15^ The full sample was used for all analyses except the functional and structural neuroimaging analyses, for which the imaging subsample was used. This study follows the Consolidated Standards of Reporting Trials (CONSORT) reporting guideline for randomized studies (eFigure 1 and eMethods in Supplement 2).

Patients were assessed on cognitive testing, symptoms, functional capacity, and functional neuroimaging at baseline, randomized in a 1:1:1 ratio to 1 of 3 widely prescribed antidepressants—escitalopram (336 patients), sertraline (336 patients), or venlafaxine-XR^14^ (336 patients)—and then reassessed on the same measures after 8 weeks of treatment. Of the total sample, 712 patients completed treatment.

### Cognitive Testing

Cognitive performance was assessed using a standardized, computerized test battery, IntegNeuro (Brain Resource), which has established norms across 9 decades of the healthy lifespan,^16^ test-retest reliability,^17^ construct validity with respect to traditional neuropsychological batteries and brain measures,^18,19^ and utility in distinguishing cognitive impairments in psychiatric groups.^20,21,22,23^ IntegNeuro assessed 9 cognitive domains (and tests), including sustained attention (Continuous Performance Test), cognitive flexibility (Stroop), decision speed (Choice Reaction Time), executive function (Maze), information processing speed (Switching of Attention), psychomotor response speed (Motor Tapping), response inhibition (Go/No-Go), verbal memory (Verbal Learning and Memory), and working memory (Digit Span). Performance was expressed as a standard deviation score referenced to a healthy norm mean of zero for each test and quantified by accuracy, reaction time, and/or completion time. Composite scores were obtained by averaging performance on each test within a domain (eTable 2 in Supplement 2).

Depressive symptom severity was assessed using criterion standard scales, the 17-item Hamilton Rating Scale for Depression (HRSD-17) with masked clinician ratings and 16-item Quick Inventory of Depressive Symptomatology–Self-Report (QIDS-SR-16)^24^ for patient reports. Function was assessed using the Social and Occupational Functioning Assessment Scale (SOFAS)^25^ on a scale of 0 to 100.

### Functional Neuroimaging

The imaging subsample underwent functional magnetic resonance imaging using 3.0 Tesla Signa HDx (GE Healthcare) with an 8-channel head coil during an established cognitive Go/No-Go task,^12,26^ engaging an equivalent domain of cognitive control as the cognitive battery (eMethods in Supplement 2). Blood-oxygen-level–dependent contrast functional images were acquired with echo-planar T2-weighted imaging (repetition time, 2500 ms; time to echo, 27.5 ms; matrix, 64 × 64; field of view, 24 cm; flip angle, 90 degrees; 40 slices, 3.5 mm–slice thickness, 123 volumes). The cognitive task robustly engages the dorsolateral prefrontal (dLPFC) and dorsal anterior cingulate (dACC) regions that define the cognitive control circuit.^27^ DLPFC and dACC activation, and functional connectivity between them, was quantified for each patient using our prior established method (PanLab Imaging Pipeline) and expressed as a standard deviation value relative to a healthy reference group,^27^ equivalent to our procedure for cognitive tests. A high-resolution T1-weighted structural scan was acquired for registration of functional images.

### Exploratory Measures

We included 3 additional measures of factors that might be alternative explanations for cognitive impairment: body mass index (BMI; calculated as weight in kilograms divided by height in meters squared), anxiety assessed using the Depression Anxiety Stress Scale-42 (DASS-42) anxiety subscale,^28^ and brain volume quantified from the structural scan using voxel-wise brain morphometry for 120 regions defined by the Automated Anatomical Atlas, an established procedure for iSPOT-D.^15^

Response was defined as an improvement of at least 50% on the HRSD-17 or QIDS-SR-16. We defined symptom remission as an HRSD-17 score of 7 or below or a QIDS-SR-16 score of 5 or below.

### Statistical Analysis

Analyses were undertaken in SPSS version 28 (IBM Corp). We imputed missing values that were less than 5% of the sample. Hypothesis tests were 2-tailed except for χ^2^ tests examining secondary treatment type differences. A nominal α = .05 was used for significance for all tests except symptoms, for which we used the Benjamini-Hochberg method^29^ with false discovery rate (FDR) of α = .05. This method adjusts the *P* values of the individual tests, considering the number of tests conducted and the expected proportion of false positives. We calculated effect sizes as Cohen *d.*

#### Deriving the Cognitive Biotype

Data-driven clustering was used to identify a cognitive biotype based on impaired cognitive performance. Expanding on a prior preliminary approach,^30^ the 9 cognitive composite scores were entered into a k-means clustering algorithm, generating 1 through 10 cluster solutions. The optimal solution was selected by convergence across multiple criteria: (1) scree plot elbow method using sum of squared Euclidean distances, (2) silhouette metric, and (3) clusters differ on a maximum number of inputs. We validated the cluster solution for clinical and mechanistic meaning using symptom, functional, neural circuit, and treatment outcome measures not used as inputs to generate the clusters.

#### Depressive Symptoms, Functional Capacity, Neural Circuit Function, and Other Considerations

Cognitive biotypes (positive and negative) were compared on overall pretreatment depressive symptom severity, individual depressive symptoms, functional capacity, neural circuit function, and exploratory BMI, anxiety, and brain volume metrics using independent sample *t* tests where the independent variable was the cognitive biotype and the dependent variable is the depressive symptom severity. We also compared clusters on individual symptoms using independent sample *t* tests corrected for multiple testing to determine whether the cognitive biotype is specifically associated with particular depressive symptoms.

#### Treatment Outcomes

χ^2^ tests were used to compare clusters on binary response and remission with antidepressant treatment and type of treatment at 8 weeks. Clusters were compared on change in function and cognitive measures using mixed model analysis of variance.

#### Treatment Outcomes as a Function of Cognitive Impairment

To further assess clinical meaningfulness of the cognitive biotype, we undertook mediation analyses to test whether symptom and functional improvement following 8 weeks of treatment rely on improvement in cognition at 8 weeks. Mediation models were implemented using the Preacher-Hayes bootstrapping method for estimating a simple mediation model with a binary predictor *X*, a continuous mediator *M*, and outcome variable *Y*. PROCESS Macro was used to implement the Preacher-Hayes method in SPSS^31^ (with *X* being cognitive biotype; *M*, change in executive function or response inhibition; *Y*, change in HRSD-17 symptom rating or SOFAS; confidence interval based on 5000 bootstrapped samples) (eMethods in Supplement 2). We note that, while temporal precedence is ideal in mediation models, the method we used does not require this to be the case. Age, baseline HRSD-17, and family history of MDD were used as covariates in the mediation models.

## Results

### Demographic Information

The 1008 MDD patients were 18 to 65 years old (mean [SD] age, 37.8 [12.6] years) and 57% female (167 [17%] Black, 83 [8%] Hispanic, 625 [62%] White). The 96 patients who participated in the imaging substudy were 47% female and had a mean age of 34.5 (13.5) years (eTable 1 in Supplement 2).

### Deriving a Cognitive Biotype

**Figure 1.**
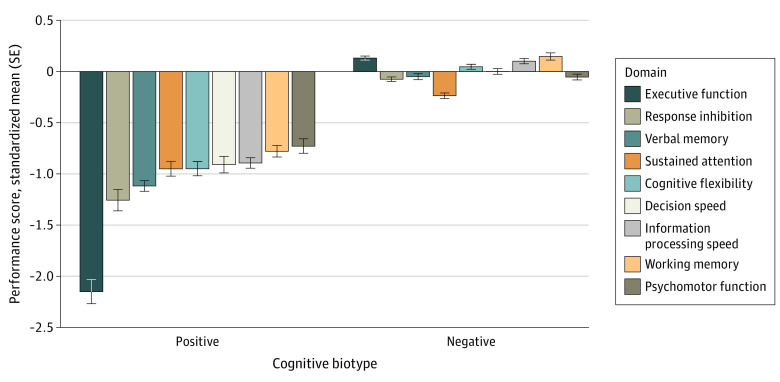
Baseline Cognitive Performance for Composite Measures in 2-Cluster Solution Standardized mean performance for baseline cognitive composite scores across 9 domains for the 2-cluster solution obtained via k-means clustering. Error bars represent the standard error of the mean. We have designated the 2 clusters cognitive biotype positive and cognitive biotype negative.

Scree plot and the silhouette metric indicated a 2-cluster solution was optimal (eResults, eFigure 2 in Supplement 2). The 2-cluster solution differed across all cognitive test scores (all *P* < .001), thus meeting our third criterion. The cluster characterized by marked impairment on all cognitive measures was present in 27% of individuals. Impairments were most pronounced for goal-directed executive function and response inhibition domains (Figure 1) relevant to the research domain criteria cognitive control construct (eTable 2 in Supplement 2). The second cluster was an intact cognitive subgroup characterized by performance well within the healthy range (Figure 1). Hereafter, we refer to these subgroups as cognitive biotype positive and cognitive biotype negative based on the rationale that the neurocognitive tests are tapping into biologically based function.

### Baseline Symptom Profiles and Neural Function

**Figure 2.**
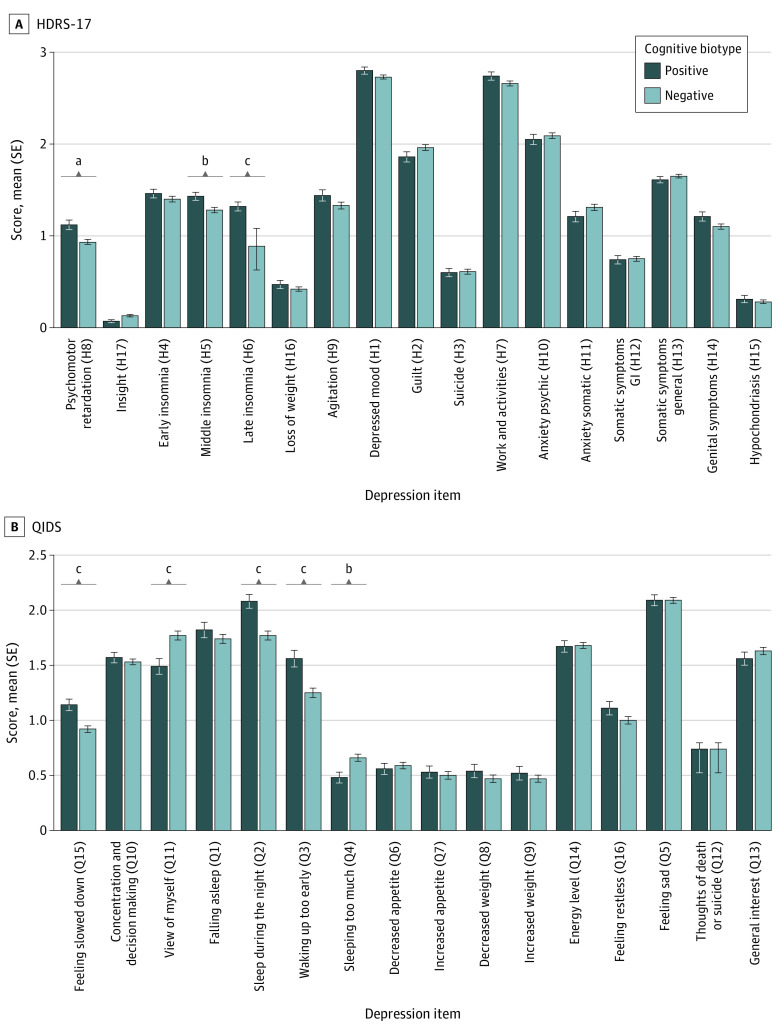
Baseline Individual Depression Scale Items Across Cognitive Biotypes Difference between cognitive biotypes on individual items of the 17-item Hamilton Rating Scale for Depression (HDRS-17) and the 16-item Quick Inventory of Depressive Symptoms–Self Report (QIDS). Significant items grouped according to symptom domain were as follows: H8 (*d* = .23; 95% CI, 0.09 to 0.37; *P* for false discovery* = *.009), Q15 (*d* = .26; 95% CI, 0.12 to 0.41; *P* for false discovery < .001), H5 (*d* = .20; 95% CI, 0.06 to 0.34; *P* for false discovery = *.*03), Q2 (*d* = .29; 95% CI, 0.15 to 0.44; *P* for false discovery *<* *.*001), H6 (*d* = .33; 95% CI, 0.19 to 0.47; *P* for false discovery *<* *.*001), Q3 (*d* = .26; 95% CI, 0.12 to 0.40; *P* for false discovery *<* *.*001), Q4 (*d* = −.22; 95% CI, −0.36 to −0.07; *P* for false discovery = *.*01), and Q11 (*d* = −.26; 95% CI, −0.40 to −0.12; *P* for false discovery *<* *.*001). Error bars represent the standard error of the mean. GI indicates gastrointestinal. ^a^*P* < .05. ^b^*P* < .01. ^c^*P* < .001.

HRSD-17 depressive symptom severity was significantly greater for the cognitive biotype positive than the negative subgroup (mean difference, 0.73; *d* = 0.18; 95% CI, 0.04 to 0.32; *P* = .01), yet this difference represented less than 1 symptom point. Clusters did not differ on overall self-reported severity on the QIDS-SR-16. Regarding individual symptoms, the cognitive biotype positive subgroup was distinguished by similar profiles on both the HRSD-17 and QIDS-SR-16, including slower information processing and effortful thinking or psychomotor retardation (HRSD-17 item 8, QIDS-SR-16 item 15), more waking during the night (HRSD-17 item 5), awaking early (HRSD-17 item 6, QIDS-SR-16 item 3), and sleeping too little (QIDS-SR-16 item 4) but significantly less self-blame (QIDS-SR-16 item 11) (Figure 2A).

**Figure 3.**
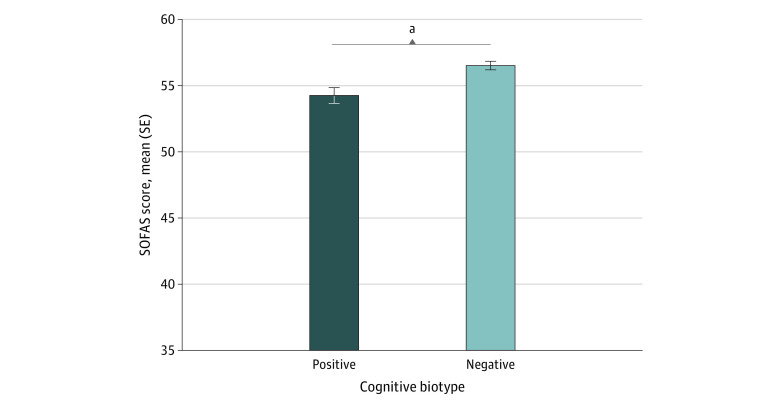
Baseline Social and Occupational Functioning Across Cognitive Biotypes Difference between cognitive biotypes on the Social and Occupational Functioning Assessment Scale (SOFAS). Error bars represent the standard error of the mean. ^a^*P* < .001.

**Figure 4.**
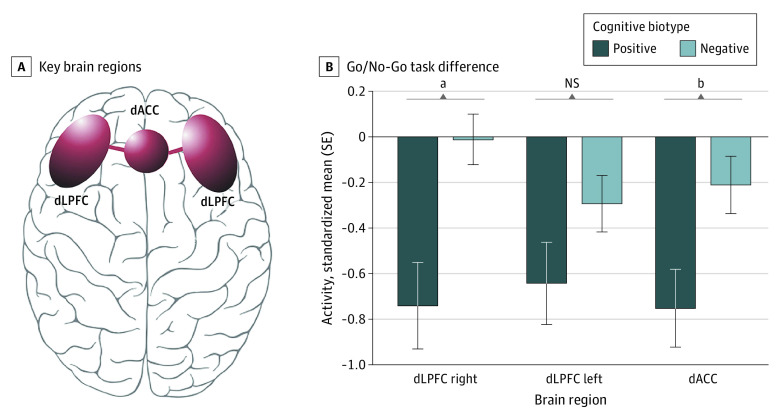
Illustration of the Cognitive Control Circuit and Baseline Task-Evoked Activation in the Cognitive Control Circuit Across Cognitive Biotypes Error bars represent the standard error of the mean. dACC indicates dorsal anterior cingulate cortex; dLPFC, dorsolateral prefrontal cortex. ^a^*P* < .05. ^b^*P* < .01.

The cognitive biotype positive subgroup had significantly greater functional impairment pretreatment compared with the negative subgroup (*d* = −0.25; 95% CI, −0.39 to −0.11; *P* < .001) as measured by the SOFAS (Figure 3). Cognitive task-evoked neural activation was significantly reduced for the cognitive biotype positive subgroup compared with the negative subgroup in right dLPFC (*d* = −0.78; 95% CI, −1.28 to −0.27; *P* = *.*003) and dACC (*d* = −0.52; 95% CI, −1.02 to −0.02; *P* = .04) regions of the cognitive control circuit (Figure 4). We did not find any meaningful differences between clusters in anxiety severity, BMI, or structural brain volume (eResults in Supplement 2).

### Cognitive Biotype and Treatment Outcomes at 8 Weeks

The cognitive biotype positive subgroup was distinguished by significantly lower rates of response and remission to antidepressants (HRSD-17 response, 103 of 188 [54.8%] vs 340 of 524 [64.9%]; *P* = .02; HRSD-17 remission, 73 of 188 [38.8%] vs 250 of 524 [47.7%]; *P* = .04). This biotype also had significantly lower response and remission rates for sertraline in particular (HRSD-17 response, 31 of 64 [48.4%] vs 132 of 182 [72.5%]; *P* < .001; HRSD-17 remission, 23 of 64 [35.9%] vs 91 of 182 [50.0%]; *P* = .04). Subgroups did not differ in profiles of individual symptom improvement within response and remission categories. Regarding functional impairment posttreatment, there was no interaction of the cognitive biotype with pretreatment vs posttreatment change in psychosocial function as measured by SOFAS. For cognitive impairment posttreatment, there was a significant interaction of cognitive subtype with pretreatment vs posttreatment change (or lack of change) in cognitive performance (executive function: *η_p_^2^* = 0.241; *P* *<* .001; response inhibition: *η_p_^2^* = 0.750; *P* < .001). Cognitive impairments persisted posttreatment in the cognitive biotype positive subgroup, remaining at least 0.2 standard deviations below the healthy mean, whereas performance improved during the treatment period for the biotype negative subgroup, especially for cognitive control domains of executive function and response inhibition (eFigure 3 in Supplement 2).

### Cognitive Impairment and Overall Symptom Response Following 8 Weeks of Treatment

**Figure 5.**
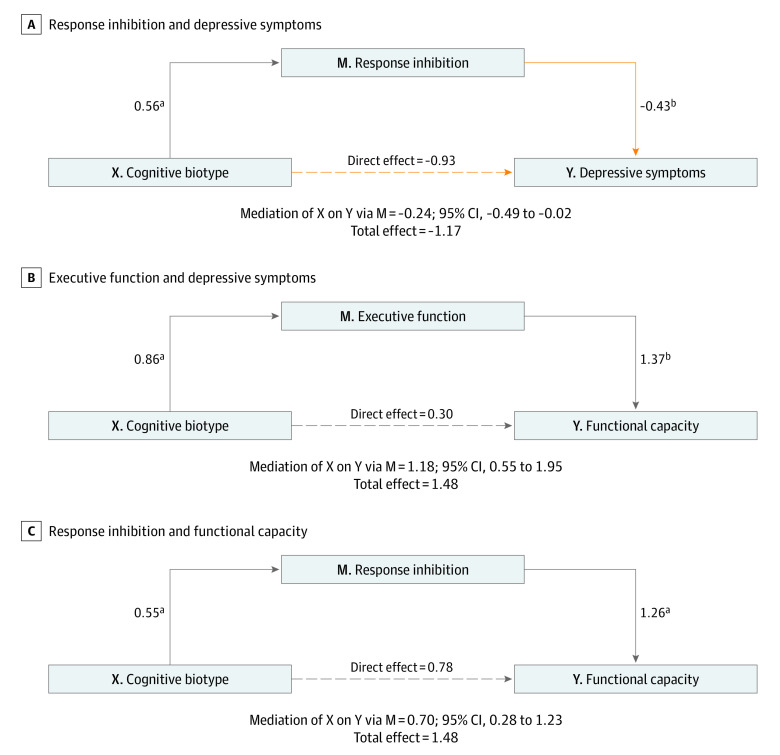
Mediation Models for Response Inhibition and Executive Function Using Depressive Symptoms and Functional Capacity as Outcomes Depressive symptoms were measured using the 17-item Hamilton Rating Scale for Depression and functional capacity using the Social and Occupational Functioning Assessment Scale. Coefficients are unstandardized. Gray arrows indicate positive associations; orange arrows, negative associations. ^a^*P* < .001. ^b^*P* < .05. ^c^*P* < .10.

Mediation models focused on executive function and response inhibition because they were most significantly impaired in the cognitive biotype positive subgroup. Lack of change in cognitive control performance was a significant mediator of the association between pretreatment cognitive biotype and lack of overall symptom relief following treatment (indirect effect a × b = −0.24; bootstrapped 95% CI, −0.49 to −0.02) (Figure 5A), but the reverse was not true. This mediator significantly contributed to the total effect model (*t* = −2.09; β = −1.17; *P* = .03) but there was no direct effect between cognitive biotype and symptom relief (*t* = −1.63; *P* = .10). See eResults in Supplement 2 for further discussion of mediation results.

## Discussion

Overall, our multimodal findings suggest the presence of a cognitive biotype of depression that represents about a quarter of all depressed patients and is characterized by prominent impairments in 2 domains of cognitive control (executive function and response inhibition). This cognitive biotype has greater severity in baseline symptoms of slowed information processing and insomnia, poor psychosocial function, reduced neural function of the brain’s cognitive control circuit, and poorer outcomes following first-line antidepressant treatment. The extent of cognitive control impairment also mediated the extent of (or lack of) symptom and psychosocial improvement posttreatment.

The identification of a distinctive cognitive biotype identified by a machine learning clustering algorithm highlights the value of disentangling the heterogeneity of depression to understand the source of mean cognitive impairments within the diagnosis of depression as a broad category. This biotype might be a major contributor to prior mean observations of impaired executive function and response inhibition within the depression diagnosis compared with healthy peers.^32^

Adding further to the value of disentangling heterogeneity in depression, the cognitive biotype identified in this study was distinguished more by a specific profile of individual symptoms than by overall depression severity. This biotype was characterized by slowed thinking (which could be anticipated given the cognitive focus of this biotype) as well as sleep problems, and aligns with prior observed associations between depression, executive function, and sleep.^33^ On psychosocial ratings, the cognitive biotype was further distinguished by poor social and occupational function, adding specificity to evidence that cognitive impairment is a major contributor to disability in depression.^1^

Our observation that the cognitive subtype was characterized by reduced activation of the dLPFC and dACC within the cognitive control circuit suggests a distinct neural mechanistic process underlying this biotype. Reduced activation in these prefrontal regions aligns with the prior proposed cognitive control biotype based on a synthesis of neuroimaging findings^13^ and the role of these regions in cognitive control performance. A unique feature of our data set was the opportunity to assess the impact of the specific cognitive biotype on outcomes following treatment. This cognitive biotype had low rates of both symptom response and remission following antidepressants, particularly for treatment with sertraline. Posttreatment, the cognitive biotype also showed continued cognitive control impairment and less improvement in psychosocial function regardless of symptom change. In mediation models, we demonstrated that less improvement in cognitive control specifically mediated the relationship between pretreatment biotype and extent of posttreatment relief from depressive symptoms. Similarly, less change in cognitive control performance mediated less improvement in psychosocial function following treatment. These mediation findings suggest that for a substantial minority of depressed patients it is necessary to improve cognition in order to improve overall depressed mood and function. The findings challenge the prevailing assumption that cognitive impairments resolve as a secondary effect of improving overall severity.

Our findings add to the growing evidence that treatments specifically targeting cognitive impairment and its mechanisms in depression are urgently needed.^34,35^ Only 1 FDA-approved antidepressant—vortioxetine—has shown promise for improving cognition^36^; however, the mechanisms underlying vortioxetine’s cognitive-enhancing effects are unknown. Future studies should utilize biomarker designs to evaluate whether outcomes for the cognitive biotype are enhanced when matched to a treatment with mechanisms targeting cognitive control circuitry and behaviors.

### Limitations

Our findings must be considered in the context of several potential limitations. First, although we rule out disorders and other factors that could affect cognitive impairment, it remains possible that other as yet unidentified behavioral or neurobiological factors contribute to the cognitive biotype. Second, although our sample was representative of multiple racial and ethnic backgrounds and clinical settings, the findings must be evaluated for generalizability. Third, the pragmatic biomarker design of the trial focused on 3 commonly prescribed antidepressants and the relevance of our approach to other antidepressants and treatment modalities requires investigation. Finally, within the mediation models, we acknowledge some overlap in measures used to allocate *X* and assess change in *M*, but not to the extent of a confounder. Future studies are warranted to test stronger causal inferences in a design optimized by experimental manipulation of cognitive biotype (*X*) and through establishing an explicit temporal precedence between *M* and *Y*. Our putative mediator, cognitive change, is suitable to such designs, as it is amenable to both measurement and manipulation. Likewise, we also acknowledge there is some circularity in using the Go/No-Go task to both generate the cognitive biotype categories (positive and negative) and link the biotypes to Go/No-Go–evoked brain response.

## Conclusions

To our knowledge, this is the first study to combine biological and nonbiological measures to verify a distinct, clinically actionable cognitive biotype of depression. If the prevalence of cognitive deficits in our sample is generalized to the US population, then approximately 5.7 million individuals with depression have cognitive impairments (27% of 21 million). Incorporating neural circuit measures enables a deeper understanding of the neurobiological mechanisms that underpin these cognitive impairments and has implications for precision treatment approaches in future studies and in practice. Biomarker trials targeting the cognitive biotype with more selective treatment strategies are urgently needed.

## Notes

Supplement 1.
Trial Protocol and Statistical Analysis Plan

Supplement 2. **eMethods.** Supplemental Methods **eResults.** Supplemental Results **eTable 1.** Demographic and Clinical Characteristics of Participants at Baseline **eTable 2.** Neurocognitive Domains, Tests, and Descriptions **eFigure 1.** CONSORT Diagram **eFigure 2.** Scree Plot of the k-means Clustering Solutions with Different Numbers of Clusters **eFigure 3.** Posttreatment Cognitive Performance for Composite Measures in Cognitive Biotypes
eReferences.

Supplement 3.
Data Sharing Statement
