# Errors in Title, Abstract, and Results

**DOI:** 10.1001/jamanetworkopen.2023.26241

**Published:** 2023-07-12

In the Original Investigation titled “A Cognitive Biotype of Depression and Symptoms, Behavior Measures, Neural Circuits, and Differential Treatment Outcomes: A Prespecified Secondary Analysis of a Randomized Clinical Trial,”^1^ published June 15, 2023, there were errors in the framing of results appearing in the Abstract and Results section. Results for the persistence of cognitive impairments regardless of symptom change were rephrased to include backing data that they remained at least 0.2 standard deviations below the healthy mean. In addition, a mistake in the title was fixed. Instead of “A Cognitive Biotype of Depression and Symptoms, Behavior Measures, Neural Circuits, and Differential Treatment Outcomes,” it should have read, “A Cognitive Biotype of Depression Linking Symptoms, Behavior Measures, Neural Circuits, and Differential Treatment Outcomes.” This article has been corrected.^1^
