# Supplementary material for: A Cognitive Biotype of Depression and Symptoms, Behavior Measures, Neural Circuits, and Differential Treatment Outcomes: A Prespecified Secondary Analysis of a Randomized Clinical Trial
Source: JAMA Netw Open. 2023 Jun 15;6(6):e2318411. doi: 10.1001/jamanetworkopen.2023.18411 (PMC10273022; doi:10.1001/jamanetworkopen.2023.18411)
Supplement: Supplement 1. — Trial Protocol and Statistical Analysis Plan [file jamanetwopen-e2318411-s001.pdf]

**Study Title:**	International Study to Predict Optimised Treatment - in Depression
**Sponsor:**	BRC Operations Pty. Ltd.<br>Level 12; 235 Jones Street<br>Ultimo, NSW 2007<br>+61 (0)2 9211 7210
**Indication:**	Major Depressive Disorder (MDD)
**Protocol ID:**	iSPOT-D
**Original Protocol:**	7 March 2008
**Incorporating Amendment 1:**	12 June 2008
**Incorporating Amendment 2:**	19 January 2009

TABLE OF CONTENTS

1.0 INTRODUCTION ..... **_3_**
1.1 Rationale 3
1.2 Objectives 4
2.0 METHODS ..... **_5_**
2.1 Trial Design and Overview 5
2.2 Subject Numbers and Recruitment 6
3. PARTICPANT RECRUITMENT ..... **_6_**
3.1 Inclusion Criteria for MDD Subject 6
3.2 Exclusion Criteria for MDD Subjects 6
3.3 Inclusion Criteria for Control Subjects 7
3.4 Exclusion Criteria for Control Subjects 7
4. STUDY PROCEDURES ..... **_8_**
4.1 Pre-treatment Procedures 8
4.2 Clinical Monitoring and Follow-up Assessments on Day 4 and Weeks 2, 4, 6, 12, 16, 24 and 52 9
4.3 Week 8 Procedures 9
4.4 Study Time Commitment 10
4.5 Summary of Procedures and Assessments 11
5. RANDOMIZATION ..... **_12_**
5.1 Treatment and Titration 12
6. CRITERIA FOR DISCONTINUATION ..... **_12_**
7. SAFETY MONITORING AND REPORTING ..... **_13_**
7.1 Assessment of Adverse Events 13
7.2 Serious Adverse Events 14
7.3 Serious Adverse Event Reporting Requirements 14
8. EHICAL CONSIDERATIONS ..... **_15_**
8.1 Good Clinical Practice 15
8.2 Institutional Review Board (IEB) / Independent Ethics Committee (IEC) Approval 15
8.3 Informed Consent 15
8.4 Confidentiality 15
8.5 Study Files and Retention of Records 16
8.6 Case Report Forms 16
8.7 Drug Accountability 16
8.8 Inspections 16
8.9 Protocol Compliance 16
9.0 SPONSOR RESPONSIBILITIES ..... **_17_**
9.1 Protocol Modifications 17
9.2 Study Report and Publication(s) 17
9.3 Joint Investigator / Sponsor Responsibilities 17
10 OBJECTIVE MARKERS AND ANALYSES ..... **_17_**
10.1 Objective Markers 17
10.2 Hypotheses 18
10.3 Sample Size and Power 22
10.4 Data Reduction 22
10.5 Planned Analyses 25
11. REFERENCES ..... **_32_**
12. PROTOCOL APPROVAL ..... **_36_**
13. INVESTIGATOR SIGNATURE PAGE ..... **_37_**
14. APPENDICES ..... **_38_**
Appendix A: The Standardised Integrative Methodology used in iSPOT 38
Appendix B: The Neuropsychology (Cognition) Test Battery 39
Appendix C: The Electrical Brain Function Test Battery 43
Appendix D: MRI and fMRI 44
Appendix E: Markers for first pass analysis 45

1.0 INTRODUCTION

Major Depressive Disorder (MDD) is a highly prevalent disorder, with lifetime prevalence reported at 16.2% in a US epidemiological survey (Kessler et al., 2003). In terms of burden of disease it is among the leading causes of disability worldwide.

There are a range of medications offered to subjects with MDD. Randomised controlled trials of acute treatment have demonstrated the efficacy of several classes of antidepressants; SSRIs, SNRIs, NRIs, MAOIs and TCAs. Around 30-45% of MDD subjects typically attain remission (defined by research criteria using objective rating scales). However, the majority of subjects in these trials do not attain remission, meaning that they are at risk for chronic depression and other morbidity factors, including suicide, substance abuse and serious medical conditions. Currently, this information can not readily be applied to practical decisions about which subject will respond best to which treatment in the clinical setting.

The crucial challenge for the clinician is the identification of markers which will predict response to individual treatments and improve subject outcome. These markers will provide an objective way to determine the magnitude of improvement that can be expected with a particular treatment for a particular individual. With the increasing focus on genetics, brain imaging and cognitive testing, there are promising findings regarding candidate markers of MDD and for treatment response in MDD. These findings provide the rationale for the current trial, that distinct combinations of these markers will predict response to individual treatment in MDD.

Questions of treatment prediction are now being addressed in 'practical trials' or 'effectiveness trials', which differ from traditional randomised, double-blind, placebo controlled efficacy trials in several ways (March et al., 2005; Tunis et al., 2003). Effectiveness trials focus on 'Personalised Medicine' (Gordon et al., 2007), which is being aggressively promoted by the FDA. These trials include a broad spectrum of subjects, compare between active treatments rather than treatment versus placebo, and focus on objective markers and their personalised real-world significance. These trials are often undertaken with self-declared subjects and diagnoses confirmed by clinicians, in outpatient settings where most depressed subjects receive treatment. Thus, effectiveness trials may be considered ecologically valid in terms of mirroring real clinical practice as much as possible.

STAR*D (Sequenced Treatment Alternatives to Relieve Depression) was a landmark example of the new effectiveness trials which have a Personalised Medicine goal. It was undertaken in primary care sites and assessed acute and long term outcomes in relation to individualised treatment. Genetic information was acquired to determine objective marker predictors of treatment outcome (Insell, 2006; Rush et al., 2006).

iSPOT-D is an effectiveness trial which has the primary aim of identifying genetic, brain structure/function and cognitive markers (or combinations of markers) which predict drug treatment response or non-response in MDD. The secondary aim is to identify which of these combinations of markers distinguish MDD from healthy subjects, and to determine whether markers of MDD overlap with markers of treatment prediction in MDD. A tertiary aim is to determine whether markers of acute treatment prediction are also predictive of functional outcome over 6-12 month follow-up periods. Subjects will include 2,016 adult patients with nonpsychotic MDD (672 in each of three drug treatment groups), who are broadly representative of this population and 672 matched healthy controls. Drug treatments were selected to reflect the most commonly used medications in two classes, namely: two SSRIs (Escitalopram and Sertraline) and a SNRI (Venlafaxine XR). Testing will occur at around 20 sites internationally (USA, Canada, UK, South Africa, New Zealand, The Netherlands and Australia).

The trial protocol has been established according to CONSORT (Consolidated Standards of Reporting Trials) guidelines where relevant to the effectiveness trial design of this trial.

1.1 Rationale

iSPOT-D is the largest marker study ever undertaken in MDD and has attracted strong interest from the NIMH and the FDA. MDD is projected to cause the second greatest global burden of disease by 2020, highlighting the urgent need for valid predictors of effective treatment response. Currently, there are no

accurate predictors of response to antidepressants in MDD, and successful treatment relies greatly on 'trial and error'.

The aim of this study is to identify genetic, brain and cognitive markers (or combinations of markers) that predict specific response to the three most commonly used antidepressants (Escitalopram, Sertraline and Venlafaxine XR,) in subjects diagnosed with major depressive disorder (MDD). This ground breaking study, has the potential to change the way in which Personalised Medicine is implemented in depression.

1.2 Objectives

The overall objectives of the iSPOT-D trial are to use standardised genetic-brain-cognition protocols to:

1. Identify markers of MDD as a diagnostic group and its subtypes; specifically:

   a. To identify genetic, brain function, brain structure, psychological* and cognitive markers (or combination of markers) of MDD versus healthy controls.

   b. To identify markers (or combination of markers) of MDD sub-types. These include the subtypes of Melancholia (versus Non-Melancholia), Atypical MDD, Anxiety, Anhedonia and Sleep.

2. Identify markers which change with acute (8 weeks) drug treatment in MDD; specifically:

   a. To identify which markers (or combination of markers) 'normalise' with SSRI or SNRI drug treatments in MDD. Change is defined by a significant shift in the direction of normal control performance.

   b. To determine whether the genetic-brain-cognition function markers (or combination of markers) 'normalise' with drug treatment in MDD (and the presumed lack of change of brain structure and genotype), and differ according to type of treatment (SSRI or SNRI).

   c. To determine if the above changes with treatment in MDD are distinct from any normative change in these markers that may occur as a function of time (8 weeks) in healthy controls.

3. Identify predictors of treatment response in MDD, and types of response; specifically:

   a. To identify pre-treatment genetic-brain-cognition function markers assessed at baseline which predict treatment **response** to acute treatment with SSRI and SNRI drugs. Treatment response is defined as a >50% decrease from baseline score on the primary and secondary endpoints. Primary endpoint is determined by the Hamilton Rating Depression Scale (HAM-D[21]). The Secondary endpoint is assessed by the Quick Inventory of Depressive Symptoms; QIDS-SR) at the end of the 8-week treatment period.

   b. To identify which pre-treatment markers (or combination of markers) assessed at the baseline visit predict **'potential placebo response'** to acute treatment with SSRI or SNRI. Potential placebo response is defined as a >50% decrease from baseline score on the secondary endpoint (QIDS- SR) within 4 days of medication.

   c. To identify which markers (or combination of markers) assessed at the pre-treatment baseline visit predict **asymptomatic status,** following acute treatment with SSRI and SNRI medication, defined as no diagnosis of depression (assessed using the MINI Plus International Neuropsychiatric Schedule; MINI PLUS; Sheehan et al., 1998; a score of < 7 on the HAM-D[21] and $\leq$ 5 on the QIDS-SR). Asymptomatic status will be compared to fully symptomatic status, defined as a diagnosis of depression (MINI PLUS) and score of $\geq$ 16 on the HAM-D[21] (and > 11 on the QIDS-SR) as outlined in Keller, 2003 and http://www.ids-qids.org.

---

* Psychological refers to measures such as exposure to early life stressors and quality of life.

d. To identify which markers (or combination of markers) assessed at pre-medication baseline predict **full remission versus relapse**, following acute treatment with SSRI and SNRI drugs, defined as asymptomatic status for at least two weeks and up to 6 months. Full remission will be compared to those with a relapse defined by fully symptomatic status for $\geq$ 2 weeks within this period.

e. To identify which markers (or combination of markers) assessed at the pre-treatment baseline predict **recovery versus recurrence**, following acute treatment with SSRI and SNRI drugs, defined as asymptomatic status for $\geq$ 6 months. Recovery will be compared to those with recurrent MDD, defined as a new episode of fully symptomatic status at $\geq$ 6 months.

f. To identify which markers (or combination of markers) assessed at the pre-treatment baseline predict **degrees of remission and recovery**, following acute treatment with SSRI and SNRI medication, assessed in terms of those with no residual symptoms versus those with > 1 residual symptoms at mild severity.

g. To identify which markers (or combination of markers) assessed at the pre-treatment baseline predict **degree of improvement in functional outcome**, following acute treatments with SSRI and SNRI drugs, assessed in terms of psychosocial functioning and quality of life.

h. To identify which markers (or combination of markers) assessed at the pre-treatment baseline predict **degrees of remission and recovery defined with dual criteria**, following acute treatment with SSRI and SNRI drugs, assessed in terms of those with no residual symptoms AND no functional impairment versus those with $\geq$1 residual symptoms at mild severity or greater and MINI Plus impairment.

i. To identify which markers (or combination of markers) assessed at the pre-treatment baseline predict the **time taken to reach remission and recovery**, following acute treatment with SSRI and SNRI medication, defined in terms of both symptoms and functional outcome.

4. To determine whether distinct individual characteristics in MDD subjects predict degree of response to different treatment with different medications; specifically:

a. To identify sub-groups of subjects defined according to their profile of clinical features (eg, sub-type) and genetic, brain function, brain structure, psychological and cognitive markers (or combination of markers) assessed at the pre-treatment baseline which predict **response** to acute treatment with SSRI and SNRI medication, defined as a >50% decrease from baseline score on the primary and secondary endpoints (HAM-D and QIDS-SR, respectively) at the end of the 8-week treatment period.

Secondary questions will also be explored systematically within each of the above objectives. For instance:

1. Whether the markers of MDD and its sub-types also distinguish clusters of comorbid conditions in MDD.

2. Whether the extent of change in markers with treatment is associated with other subject's characteristics, such as age and sex.

3. If markers which predict response and severity to treatment, also predict other aspects of drug response, such as number of side effects.

2.0 METHODS

2.1 Trial Design and Overview

This is an open-label, randomised (effectiveness) study (i.e. comparison of active treatments) to identify genetic markers, brain function, brain structure, and psychological and cognitive indicators (or a combination of markers) in MDD subjects versus healthy controls. Approximately 2,016 subjects with

major depressive disorder (MDD) across multiple international sites (USA, Canada, UK, South Africa, New Zealand, The Netherlands and Australia) will be randomised to one of three approved and effective treatment arms:

- Treatment A Escitalopram
- Treatment B Sertraline
- Treatment C Venlafaxine XR.

A group of matched healthy controls (n = 672) will also be enrolled.

To be eligible for screening, subjects must provide written informed consent, be 18-65 years of age and English or Dutch speaking. Subjects diagnosed with MDD must meet DSM-IV criteria for MDD (without evidence of suicidal ideations and/or tendencies of bipolar disorder, psychosis or primary eating disorder), have an HAM-D[21] score $\geq$ 16 and must have no recent use of antidepressant medication, including protocol defined medications. Subjects with Anxiety Disorders as a secondary diagnosis (GAD, Specific Phobias, Agoraphobia and Panic Disorder) are eligible to be screened.

Healthy control subjects will be matched for age, gender and years of education to enrolled MDD subject and must not have evidence of an Axis 1 disorder (including a known family history of psychiatric or neurological illness). Please see section 3.1 for a full list of inclusion/exclusion criteria.

As part of the study, subjects will be seen in the clinic at least twice; at Pre-treatment and again at Week 8 post treatment. Screening and Baseline procedures/assessments may be completed on different days, however all screening and Baseline procedures/assessments must be completed with 48-hours of each other and prior to first dose of medication. The protocol defined follow-up assessments/monitoring will be completed via telephone and the internet. During the follow-up assessments, subjects who have discontinued treatment will be classified as treatment failures, and not replaced.

2.2 Subject Numbers and Recruitment

Approximately 2,016 MDD subjects (672 for each of the three treatment groups) and a group of 672 matched healthy controls will be recruited from equivalent demographic and geographic areas for a total number of subjects of 2,688.

MDD subjects may be recruited from both out patient and in-patient referrals and will be identified via primary and psychiatric care settings.

Inclusion and exclusion criteria are outlined below.

3. PARTICPANT RECRUITMENT

3.1 Inclusion Criteria for MDD Subject

1. Meet DSM-IV criteria for primary diagnosis of MDD (as determined by a psychiatrist, general practitioner or clinical psychologist in conjunction with the clinical work-up undertaken by trained research assistants.

2. HAM-D[21] score of $\geq$ 16.

3. 18-65 years age-range (inclusive), to avoid depression resulting from age-related brain pathologies (e.g. white matter hyper-intensities, Rogers et al., 1998).

4. Subjects who are fluent and literate in English or Dutch.

5. Written, informed consent.

3.2 Exclusion Criteria for MDD Subjects

1. Presence of suicidal ideations and/or tendencies (as determined by a score $\geq$ 8 on Section C (Suicidality) of the MINI Plus), Bipolar I-III, psychosis, primary eating disorders, Post

Traumatic Stress Disorder (PTSD), Obsessive Compulsive Disorder (OCD), as well as any Axis II personality disorders.

2. Pregnancy and women of child bearing potential who are not taking a medically accepted form of contraception and are at risk of becoming pregnant during the study.

3. Breastfeeding.

4. Known contra-indication to the use of Escitalopram, Sertraline or Venlafaxine XR as defined in the product package insert for each drug (including previous treatment failure at the highest recommended dose).

5. Use of any antidepressant or CNS drug which can not be washed out prior to participation.

6. Use of any medication which is known to be contraindicated with Escitalopram, Sertraline, or Venlafaxine XR (refer to the product package insert for each drug).

7. Known medical condition, disease or neurological disorder which might, in the opinion of investigator/s, interfere with the assessments to be made in the study or put subjects at increased risk when exposed to optimal doses of the drug treatment (including, but not limited to: a cardiac rhythm disorder, prior myocardial infarction, angina, congestive heart failure, hypertension, stroke, active peptic ulcer, renal insufficiency, liver disease, neoplastic disease, inflammatory disease, diabetes, blood clotting disorder).

8. Personal history of physical brain injury or blow to the head that resulted in loss of consciousness of greater than five minutes.

9. Recent/current substance dependence (including alcohol equalling 29 standard alcoholic drinks per week for males; > 15 for females) in the past six months.

10. Participation in an investigational study within four months of the baseline visit (excluding follow-up studies in which the test drug/device has been registered in a major market) in which subjects have received an experimental drug/device that could affect the primary end points of this study.

11. Subjects who, in the opinion of the investigator, have a severe impediment to vision, hearing and/or hand movement, which is likely to interfere with their ability to complete the test batteries.

12. Subjects who, in the opinion of the investigator, are unable and/or unlikely to comprehend and follow the study procedures and instructions.

3.3 Inclusion Criteria for Control Subjects

1. 18-65 years age-range (inclusive), to avoid depression resulting from age-related brain pathologies (e.g. white matter hyper-intensities, Rogers et al., 1998).

2. Subjects who are fluent and literate in English or Dutch

3. Written, informed consent.

3.4 Exclusion Criteria for Control Subjects

1. Current or previous diagnosis of MDD, presence of suicidal ideations and/or tendencies (as determined by a score ≥ 8 on Section C (Suicidality) of the MINI Plus), Bipolar I-III, psychosis, primary eating disorders, Post Traumatic Stress Disorder (PTSD), Obsessive Compulsive Disorder (OCD), as well as any Axis II personality disorders..

2. Pregnancy and women of child bearing potential who are not taking a medically accepted form of contraception and are at risk of becoming pregnant during the study.

3. Breastfeeding.

4. Known medical condition, disease or neurological disorder which might, in the opinion of investigator/s, interfere with the assessments to be made in the study (including, but not limited to: a cardiac rhythm disorder, prior myocardial infarction, angina, congestive heart failure, hypertension, stroke, active peptic ulcer, renal insufficiency, liver disease, neoplastic disease, inflammatory disease, diabetes, blood clotting disorder).

5. Personal history of physical brain injury or blow to the head that resulted in loss of consciousness of greater than five minutes.

6. Recent/current substance dependence (including alcohol equalling 29 standard alcoholic drinks per week for males; > 15 for females) in the past six months.

7. Participation in an investigational study within four months of the baseline visit (excluding follow-up studies in which the test drug/device has been registered in a major market) in which subjects have received an experimental drug/device that could affect the primary end points of this study.

8. Subjects who, in the opinion of the investigator, have a severe impediment to vision, hearing and/or hand movement, which is likely to interfere with their ability to complete the testing batteries.

9. Subjects who, in the opinion of the investigator, are unable and/or unlikely to comprehend and follow the study procedures and instructions.

4. STUDY PROCEDURES

Screening and Baseline procedures/assessments may be completed on different days, however all pre-treatment procedures/assessments must be completed with 48-hours of each other and prior to first dose of medication.

4.1 Pre-treatment Procedures

The following will be performed:

1. Obtain informed consent. The research assistant or trial coordinator/research staff will explain the study and ascertain potential subject willingness to enter the study prior to any other procedure/assessment being completed.
2. Collect concomitant medications (prescription and over-the-counter).
3. Drug screen for illicit drug use.
4. Urine for pregnancy (females of child-bearing potential only).
5. Collection of blood samples for batched genetic analysis.
6. Psychological and clinical work-up:
    a. The Mini International Neuropsychiatric Interview (MINI Plus) to confirm MDD diagnosis against a checklist for DSM-IV criteria (for subjects).
    b. Hamilton Rating Depression Scale (HAM-D[21]).
    c. CORE Rating Scale to assess melancholia versus non-melancholia subtypes of depression
    d. Columbia Atypical Depression Diagnostic Scale (ADDS) to access atypical depression.
    e. Social and Occupational Functioning Assessment Scale (SOFAS) (DSM-IV, 1994).
7. Brain Resource Web Questionnaire:
    a. In addition to the medical history and demographic information provided by the MINI Plus listed above, detailed information will also be recorded via the Brain Resource

        Web-based questionnaires. It provides a standardised assessment of the following areas:
            i. Vision
            ii. Hearing
            iii. Handedness
            iv. Sleep History
            v. Smoking History
            vi. Alcohol History
            vii. Illicit Drug History
            viii. Early Life Stress/stressful life events
    b. Brain Resource Inventory of Social Cognition including DASS1-21 (BRISC).
    c. Depression, Anxiety and Stress Scale (DASS21-42).
    d. Neuroticism, Extraversion, and Openness Five Factor Inventory (NEO-FFI).
    e. Satisfaction with Life Scale (SWLS).
    f. Emotion Regulation Questionnaire (ERQ).
    g. Quick Inventory of Depressive Symptomatology - Self Report (QIDS-SR) for depression severity.
    h. 26-item World Health Organisation Quality of Life Scale Brief Version to measures of real-world psychosocial functioning in domains of work, social, health and general functioning.
8. Psychophysiological assessment including, heart rate, respiratory rate, sweat rate, electroencephalogram – EEG, an Event Related Potential – ERP and a Cognitive Test battery (refer to appendix A for details).
9. Structural and functional MRI (to be conducted at selected sites in 10% of subjects).
10. Subject randomization and medication.

4.2 Clinical Monitoring and Follow-up Assessments on Day 4 and Weeks 2, 4, 6, 12, 16, 24 and 52

At these additional time points (+ 3 days for Day 4 and +/- 3 days for Weeks 2-52), the following assessments will be completed over the telephone and the internet:

Subjects will be asked to login onto the BRC Web based questionnaire to complete the following:
1. Quick Inventory of Depressive Symptoms (QIDS-SR).
2. Self-Rated Global Measure of the Frequency, Intensity, and Burden of Side Effects Rating (FIBSER).

Subjects will be asked about current concomitant medication use from previous visit. MDD subjects will be asked about he randomized antidepressant medication (start and stop dates, frequency and dose) information.

4.3 Week 8 Procedures
Approximately eight-weeks following the pre-treatment assessments (+/- 3 days), all subjects will be re-tested with the full battery of assessments and procedures as described above (except for pregnancy screen).

These assessments will include:
1. Drug screen for illicit drug use.
2. Psychological Work-up
    a. HAM-D^{21}.
    b. Core Rating Scale
    c. Social and Occupational Functioning Assessment Scale (SOFAS).
3. Web Questionnaire (Web-Q), including:
    a. BRISC (including DASS$^{1-21}$)
    b. Depression, Anxiety and Stress Scale (DASS$^{21-42}$).
    c. Neuroticism, Extraversion, and Openness Five Factor Inventory (NEO-FFI).
    d. Emotion Regulation Questionnaire (ERQ).
    e. Satisfaction with Life Scale (SWLS).
    f. Quick Inventory of Depressive Symptomatology (QIDS-SR).

    g. 26-item World Health Organisation Quality of Life Scale Brief Version (The WHOQOL).
    h. FIBSER.
4. Psychophysiological assessment (including, heart-rate, respiratory rate, sweat rate, EEG and ERP) and the Cognitive test battery.
5. Repeat functional MRI, if applicable.
6. Update concomitant medication use from previous visit.
7. Antidepressant Drug accountability for MDD subjects only.

4.4 Study Time Commitment

Table 1 provides the estimated time commitment required of each subject enrolled:

Table 1

Event	APPRX. TIME (mins)
Pre-treatment (Screen and Baseline)	340
Clinical Monitoring (Day 4 and Weeks 2, 4 and 6)	Four 20-minute phone interviews + internet session
Week 8	240
Clinical Monitoring (weeks 12, 16, 24 and 52)	Four 20-minute phone interviews + internet session

4.5 Summary of Procedures and Assessments

Table 2

Measures	Day 0 Pre-Tx[d]	Day 4	Wk 2	Wk 4	Wk 6	Wk 8	Wk 12	Wk 16	Wk 24	Wk 52
Informed Consent	X									
Confirmation of Diagnosis, Severity and Sub-typing (MINI, HAM-D, ADDS, SOFAS & Core)	X					X[g]				
Blood & Urine Collection[a]	X					X[f]				
BRC Web-Questionnaire	X					X				
Randomisation/Medication Prescription	X									
Cognitive Test Battery	X					X				
Psychophysiology (EEG)	X					X				
sMRI/fMRI[c]	X					X[e]				
Drug Accountability		X	X	X	X	X	X	X	X	X
Concomitant Medications	X	X	X	X	X	X	X	X	X	X
Clinical Follow-up Assessment (QIDS & FIBSER)[b]		X	X	X	X	X	X	X	X	X

[a] Urine for toxicity screen and pregnancy test (women of child-bearing potential only).
[b] Subjects to complete online questionnaire and will be contacted via telephone
[c] Ten percent (10%) of subject will have a structural MRI completed.
[d] Pre-treatment (Pre-tx) procedures/assessments may be completed on different days, however all screening and Baseline procedures/assessments must be completed with 48-hours of each other and prior to first dose of medication.
[e] Subjects who complete the sMRI and the fMRI will repeat the fMRI at Week 8.
[f] Urine toxicity screen only at Week 8
[g] Only the HAM-D, SOFAS and Core to be repeated

5. RANDOMIZATION

Approximately 2016 subjects will be randomised in a 1:1:1 ration to receive Escitalopram 10 mg/day as a single dose, increased to max 20 mg/day, if needed; Sertraline 50 mg/day as a single dose, increased to max of 200 mg/day, if needed or Venlafaxine XR 75 mg/day given once daily; increased to 150-225 mg/day, if needed. A centralised randomization procedure will be used whereby a treatment is randomly assigned to a subject according to a predetermined randomization code.
Open-label Escitalopram, Sertraline or Venlafaxine will be supplied to the subject via a prescription. Study medication will not be supplied by the sponsor.

5.1 Treatment and Titration

Subjects will be grouped according to a randomised design in which both the subject and clinician will be in the state of "equipoise" (i.e. no treatment will be unacceptable or clearly superior, see Lavori et al., 2001 for details), which is designed to more closely mimic clinical practice.

Dosage is set according to the pharmaceutical product information. Clinicians may use a dose escalation algorithm in conjunction with symptom ratings to enhance care quality to ensure that those who can remit will remit. Subjects will be titrated to their optimal dosage over the 8 week study period, and titration details will be recorded in the CRF. Treatments for this study include:

Treatment A: Escitalopram (SSRI); 10 mg/day as a single dose, increased to max 20 mg/day, as required.
Treatment B: Sertraline (SSRI); 50 mg/day as a single dose, increased to max of 200 mg/day, as required.
Treatment C: Venlafaxine XR (SNRI); 75 mg/day given once daily; increased to 150-225 mg/day, as required.

Subjects who discontinue randomised medication or who are placed on an alternate medication may be removed from the trial but their data and associated details will be analysed.

"Medication naïve" will be defined as those subjects with no previous ingestion of antidepressants. Antidepressant free is defined as no exposure for at least 5 half lives of the product concerned. For the purposes of this study, St. Johns Wort (Hypericum) is considered an antidepressant. Subjects shall not be included in the study should they need to be washed out from any of the 3 study drugs.

6. CRITERIA FOR DISCONTINUATION

Subjects who do not meet the inclusion/exclusion criteria will be classified as Screen Failures. A list of reasons for non-inclusion will be maintained at each site and recorded in the CRFs.

Reasons for discontinuation include the following:

1. Completion of the study.
2. Withdrawal of consent by the subject (for any reason).
3. Withdrawal based on investigator decision.
4. Treatment failure requiring addition or change of medication, or addition of a concomitant medication not allowed by the protocol.
5. Withdrawal for administrative reasons (e.g. subject relocates, is unable to attend follow-up visits).
6. Withdrawal as a result of an adverse event (e.g. neurological event, side effect)
7. Significant protocol violation.

The occurrence of one of these categories will be noted in the CRF and taken into account in the final interpretation of the study results.

7. SAFETY MONITORING AND REPORTING

An adverse event (AE) is any untoward medical occurrence (including benefits) in an investigational subject administered a medicinal product and which does not necessarily have a causal relationship with this treatment. An AE can therefore be any unfavourable and unintended sign, symptom, or disease temporally associated with the use of a medicinal product, whether or not considered related to the medicinal product.

Any pre-existing condition that increases in severity, or changes in nature during or as a consequence of the treatment medication phase of a human clinical trial, will also be considered an AE.

AEs may also include any complication that occurs as a result of a protocol-associated procedure (e.g., MRI, blood draw, etc.) during or after screening (before the administration of treatment medication).

All AEs that occur after the screening visit and throughout the duration of the study should be recorded as an AE.

An AE does not include the following:
- Medical or surgical procedures (e.g., surgery, endoscopy, tooth extraction, transfusion) performed; the condition that leads to the procedure is an adverse event.
- Pre-existing diseases or conditions or laboratory abnormalities present or detected before the screening visit that do not worsen.
- Situations where an untoward medical occurrence has not occurred (e.g., hospitalization for elective surgery, social and/or convenience admissions).
- Overdose of either treatment medication or concomitant medication without any signs or symptoms, unless the subject is hospitalised for observation.

Any medical condition or clinically significant laboratory abnormality with an onset date before the screening visit and not related to a protocol-associated procedure is not an AE. It is considered to be pre-existing and should be documented as part of the medical history.

In addition, pregnancy is not considered an AE. However, any pregnancy complication or elective termination of a pregnancy for medical reasons will be recorded as an AE or an SAE.

7.1 Assessment of Adverse Events

All AEs will be assessed by the investigator and recorded on the AE CRF page. The AE entry should indicate whether or not the AE was serious, the start date (AE onset), the stop date (date of AE resolution), whether or not the AE was related to treatment medication or to a study procedure, the action taken with treatment medication due to the AE, and the severity of the AE.

The relationship to treatment medication therapy should be assessed using clinical judgment and the following definitions:

- **No**: Evidence exists that the adverse event has an aetiology other than the treatment medication. For SAEs, an alternative causality must be provided (e.g., pre-existing condition, underlying disease, intercurrent illness, or concomitant medication).
- **Yes**: A temporal relationship exists between the AE onset and administration of the treatment medication that cannot be readily explained by the subject's clinical state or concomitant therapies. Furthermore, the AE appears with some degree of certainty to be related, based on the known therapeutic and pharmacologic actions or adverse event profile of the treatment medication. In case of cessation or reduction of the dose, the AE abates or resolves and reappears upon rechallenge.

It should be emphasised that ineffective treatment should not be considered as causally related in the context of adverse event reporting.

The relationship to study procedures (e.g., invasive procedures such as venepuncture) should be assessed using the following definitions:

- **No:** Evidence exists that the adverse event has an aetiology other than the study procedure.
- **Yes:** The adverse event occurred as a result of protocol-mandated procedures such as venepuncture or biopsy.

7.2 Serious Adverse Events

A **serious adverse event** (SAE) is defined as follows:
Any adverse drug experience occurring at any dose that results in any of the following outcomes:
- Death.
- Life-threatening situation (subject is at immediate risk of death).
- In-subject hospitalization or prolongation of existing hospitalization (excluding those for study therapy or placement of an indwelling catheter, unless associated with other SAEs)
- Persistent or significant disability/incapacity.
- Congenital anomaly/birth defect in the offspring of a subject who received treatment medication.
- Other: medically significant events that may not be immediately life-threatening or result in death or hospitalization, but based upon appropriate medical and scientific judgment, may jeopardise the subject or may require medical or surgical intervention to prevent one of the outcomes listed above.

Examples of such events are as follows:
- Intensive treatment in an emergency room or at home for allergic bronchospasm.
- Blood dyscrasias or convulsions that do not result in hospitalization.
- Development of drug dependency or drug abuse.

Clarification of Serious Adverse Events
- Death is an outcome of an AE, and not an adverse event in itself.
- All deaths, regardless of cause or relationship, must be reported for subjects on study and for deaths occurring within 30 days of last treatment medication dose or within 30 days of last study evaluation, whichever is longer.
- "Occurring at any dose" does not imply that the subject is receiving treatment medication at the time of the event. Dosing may have been given as treatment cycles or interrupted temporarily before the onset of the SAE, but may have contributed to the event.
- "Life-threatening" means that the subject was at immediate risk of death from the event as it occurred. This does not include an event that might have led to death if it had occurred with greater severity.
- Complications that occur during hospitalizations are AEs. If a complication prolongs the hospitalization, it is a SAE.
- "In-patient hospitalization" means the subject has been formally admitted to a hospital for medical reasons, for any length of time. This may or may not be overnight. It does not include presentation and care within an emergency department.
- The investigator should attempt to establish a diagnosis of the event on the basis of signs, symptoms and/or other clinical information. In such cases, the diagnosis should be documented as the AE and/or SAE and not the individual signs/symptoms.

A distinction should be drawn between seriousness and severity of AEs. An AE that is assessed as Grade 4 (potentially life-threatening) should not be confused with an SAE. Severity is a category utilised for rating the intensity of an event; and both AEs and SAEs can be assessed as Grade 4. An event is defined as "serious" when it meets one of the predefined outcomes described above in Section 7.2.

7.3 Serious Adverse Event Reporting Requirements

All Serious Adverse Events
All SAE must be record on the AE CRF and complete the "Serious Adverse Event Report" form. Brain Resource may request additional information from the investigator to ensure the timely completion of accurate safety reports. Follow-up of adverse events will continue through the last day on study (including the follow-up off-study medication period of the study) and/or until the investigator and/or Brain Resource determine that the subject's condition is stable. Brain Resource may request that certain adverse events be followed until resolution.

The investigator must take all measures necessary for resolution of the SAE. Any medications necessary for treatment of the SAE must be recorded onto the concomitant medication section of the subject's CRF.

Investigator and Sponsor Reporting Requirements for SAEs

The investigator should notify the Institutional Review Board (IRB) or Independent Ethics Committee (IEC) as soon as is practical, of serious events in writing where this is required by local regulatory authorities, and in accordance with the local institutional policy.

In accordance with the EU Clinical Trials Directive (2001/20/EC), the Sponsor or its designee will notify the Ethics Committees of the concerned Member States of serious adverse events that are unexpected and possibly attributable to the treatment medication.

8. EHICAL CONSIDERATIONS

8.1 Good Clinical Practice

The investigator will ensure that this study is conducted in accordance with the principles of the "Declaration of Helsinki" (as amended in Edinburgh, Tokyo, Venice, Hong Kong, and South Africa), International Conference on Harmonisation (ICH) guidelines, or with the laws and regulations of the country in which the research is conducted, whichever affords the greater protection to the study subject. The investigator will ensure that the basic principles of "Good Clinical Practice," as outlined in 21 CFR 312, subpart D, "Responsibilities of Sponsors and Investigators," 21 CFR, part 50, 1998, and 21 CFR, part 56, 1998, are adhered to.

8.2 Institutional Review Board (IEB) / Independent Ethics Committee (IEC) Approval

This protocol and any accompanying material to be provided to the subject (such as advertisements, subject information sheets, or descriptions of the study used to obtain informed consent) will be submitted by the investigator to an IRB/IEC Approval from the *IRB/EC* must be obtained **before** starting the study and should be documented in a letter to the investigator specifying the protocol number, protocol version, protocol date, documents reviewed, and date on which the committee met and granted the approval.

Any modifications made to the protocol after receipt of IRB/EC approval must also be submitted to the IRB/EC for approval before implementation.

8.3 Informed Consent

The investigator is responsible for obtaining written informed consent from each individual participating in this study after adequate explanation of the aims, methods, objectives, and potential hazards of the study and before undertaking any study-related procedures. The investigator must utilise an IRB/EC approved consent form for documenting written informed consent. Each informed consent will be appropriately signed and dated by the subject or the subject's legally authorised representative and the person obtaining consent.

8.4 Confidentiality

The investigator must assure that subjects' anonymity will be strictly maintained and that their identities are protected from unauthorised parties. Only subject initials, date of birth and an identification code (i.e., not names) should be recorded on any form or biological sample submitted to the sponsor, IRB/EC or laboratory. The investigator must keep a screening log showing codes, names, and addresses for all subjects screened and for all subjects enrolled in the trial.

The investigator agrees that all information received from Brain Resource, including but not limited this protocol, CRFs, the included assessments, and any other study information, remain the sole and exclusive property of Brain Resource during the conduct of the study and thereafter. This information is not to be disclosed to any third party (except employees or agents directly involved in the conduct of the study or as required by law) without prior written consent from Brain Resource. The investigator

further agrees to take all reasonable precautions to prevent the disclosure by any employee or agent of the study site to any third party or otherwise into the public domain.

8.5 Study Files and Retention of Records

The investigator must maintain adequate and accurate records to enable the conduct of the study to be fully documented and the study data to be subsequently verified. These documents should be classified into at least the following two categories: (1) investigator's study file, and (2) subject clinical source documents.

The investigator's study file will contain the protocol/amendments, CRF and query forms, *IRB/EC* and governmental approval with correspondence, informed consent, drug records, staff curriculum vitae and authorization forms, and other appropriate documents and correspondence.

Subject clinical source documents (usually defined by the project in advance to record key efficacy/safety parameters independent of the CRFs) would include (although not be limited to) the following: subject hospital/clinic records, physician's and nurse's notes, appointment book, original laboratory reports, electrocardiogram (ECG), electroencephalogram (EEG), and/or other brain assessments, *x-ray*, pathology and special assessment reports, consultant letters, screening and enrolment log, etc.

All clinical study documents must be retained by the investigator until at least 2 years after the investigation is discontinued and regulatory authorities have been notified. Investigators may be required to retain documents longer if required by applicable regulatory requirements or an agreement with Brain Resource. The investigator must notify Brain Resource before destroying any clinical study records.

Should the investigator wish to assign the study records to another party or move them to another location, Brain Resource must be notified in advance.

If the investigator cannot guarantee this archiving requirement at the study site for any or all of the documents, special arrangements must be made between the investigator and Brain Resource to store these in sealed containers outside of the site so that they can be returned sealed to the investigator in case of a regulatory audit. When source documents are required for the continued care of the subject, appropriate copies should be made for storage outside of the site.

8.6 Case Report Forms

For each subject enrolled, a CRF must be completed and signed by the principal investigator or sub-investigator within a reasonable time period after data collection. This also applies to records for those subjects who fail to complete the study (even during a pre-randomisation screening period if a CRF was initiated). If a subject withdraws from the study, the reason must be noted on the CRF. If a subject is withdrawn from the study because of a treatment-limiting adverse event, thorough efforts should be made to clearly document the outcome

8.7 Drug Accountability

The investigator or designee is responsible for ensuring adequate compliance with treatment medication. Accountability records should include date and time of first dose, and the number of missed doses.

8.8 Inspections

The investigator should understand that source documents for this trial should be made available to appropriately qualified personnel from Brain Resource or its representatives, to IRBs [or] IECs, or to regulatory authority or health authority inspectors.

8.9 Protocol Compliance

The investigator is responsible for ensuring the study is conducted in accordance with the procedures and evaluations described in this protocol.

9.0 SPONSOR RESPONSIBILITIES

9.1 Protocol Modifications

Protocol modifications, except those intended to reduce immediate risk to study subjects, may be made only by Brain Resource. All protocol modifications must be submitted to the *IRB/EC* in accordance with local requirements. Approval must be obtained before changes can be implemented.

9.2 Study Report and Publication(s)

A clinical study report will be prepared and provided to the regulatory agency(ies).
After conclusion of the study investigators in this study will be encouraged to communicate, orally present, or publish in scientific journals or other scholarly media once patent protection has been achieved (where relevant).

No such communication, presentation, or publication will include Brain Resource's confidential information. The investigator will submit any proposed publication or presentation along with the respective scientific journal or presentation forum at least 60 days before submission of the publication or presentation.

9.3 Joint Investigator / Sponsor Responsibilities

Access to Information for Monitoring

In accordance with ICH Good Clinical Practice (ICH GCP) guidelines, the study monitor must have direct access to the investigator's source documentation in order to verify the data recorded in the CRFs for consistency.

The monitor is responsible for routine review of the CRFs at regular intervals throughout the study to verify adherence to the protocol and the completeness, consistency, and accuracy of the data being entered on them. The monitor should have access to any subject records needed to verify the entries on the CRFs. The investigator agrees to cooperate with the monitor to ensure that any problems detected in the course of these monitoring visits are resolved.

Access to Information for Auditing or Inspections

Representatives of regulatory authorities or of Brain Resource may conduct inspections or audits of the clinical study. If the investigator is notified of an inspection by a regulatory authority the investigator agrees to notify the Brain Resource Medical Monitor immediately. The investigator agrees to provide to representatives of a regulatory agency or Brain Resource access to records, facilities, and personnel for the effective conduct of any inspection or audit.

Study Discontinuation

Both the sponsor and the investigator reserve the right to terminate the study at any time. Should this be necessary, both parties will arrange discontinuation procedures and notify the appropriate regulatory authority(ies), IRB/IEC. In terminating the study, Brain Resource and the investigator will assure that adequate consideration is given to the protection of the subjects' interests.

10 OBJECTIVE MARKERS AND ANALYSES

10.1 Objective Markers

From the Genetic, Cognitive and Electrical Brain Function assessments outlined above, 165 objective markers will be considered in the study. MRI markers will be available for a subset of subjects, providing complementary information to validate (converging evidence) Genetic/Cognitive/Electrical brain function outcomes.

Clinical assessments will provide information from which to determine if objective measures are able to identify subjects with MDD and predict treatment response and severity of depression, and to determine remission.

See Appendix E for the list of 165 objective markers. The analysis will follow a stepwise stratified approach, which has been shaped after consultation with the FDA.

10.2 Hypotheses
The hypotheses relate to the objectives of the trial outlined in Section 1.1.

10.2.1 Primary hypotheses for Markers of MDD.

In regard to Objective 1 (identifying markers of MDD as a diagnostic group and its sub-types), the hypotheses draw on evidence for candidate markers from previous studies which have focused on particular measures, and from reviews of the most robust candidates (eg. Hasler et al., 2004). Specific hypotheses follow on the next page.

1. MDD will be distinguished by the following profile of genetic, psychological, cognitive and brain markers:

- Greater exposure to early life stressors.
- General cognition:
  Poor verbal recall and attention/vigilance, and slowed information processing, particularly in the sensori-motor domain.
- Social cognition:
  Higher neuroticism and negativity bias in terms of both temperament and preferential recognition of negative facial emotion and poor emotional intelligence (particularly emotion regulation).
- Electrical brain function:
  *A*bnormal EEG Alpha asymmetry, together with reduced EEG Alpha power but increased Theta slow-wave power, reflecting cortical arousal dysregulation. During activation tasks, poor memory and attention reflected in reduced and slowed ERPs during working memory and selective attention tasks. In addition, a neural negativity bias reflected in reduced and slowed ERPs during emotion perception. Neural synchrony measures are predicted to show a loss of synchronization during working memory and attention tasks (reflecting slower and maladaptive integration of information) but excessive synchrony during emotion perception (reflecting excessive integration due to negativity bias).
- Concurrent arousal measures:
  Reduced autonomic arousal (hypoarousal), and poor autonomic regulation, reflected in reduced heart rate variability. Abnormal pattern of startle modulation reflecting the negativity bias (eg., excessive facilitation of negative emotion, responding to neutral as if negative, reduced to positive emotion).
- MRI grey matter and DTI:
  Reduced grey matter in limbic structures (hippocampus, amygdala) and anterior cingulate/medial prefrontal cortex In particular. Corresponding reduction in fractional anisotropy in temporal and frontal regions.
- Functional MRI:
  Enhanced activity in amygdala but reduced activity in anterior (particularly subgenual) cingulate, along with abnormal connectivity in amygdala-anterior cingulate/medial prefrontal networks. Reduced activation in hippocampus and dorsolateral prefrontal cortex during cognitive tasks.
- Genetic:
  Abnormalities in cognition, brain function and MRI will be most pronounced in MDD subjects with BDNF Met allele, 5HTT (S allele), 5HTA1 (C1019), 5HT2A (1438A/G and 102 T-C), NET (182C) and/or GABRA5 variants.
- Combination of Markers:
  We will test a path model in which cognition and brain-arousal markers are most pronounced in MDD subjects with the above genetic variants as well as higher exposure to early life stress (eg. Pilot data in Figure 1). The gene-stress interaction will predict the loss of grey matter/anisotropy. Functional brain alterations will mediate the alterations in cognition. It is expected that BDNF and GABA variants will mediate general cognitive, hippocampal-dorsolateral prefrontal and autonomic arousal disturbances in particular, while 5HT variants

allele will mediate social cognition, amygdala-ACC changes and startle-related disturbances. GABA variants may contribute to alterations in neural synchrony.

Markers which distinguish MDD will be associated with severity of depressive symptomatology, assessed by the HAM-D/IDS/QIDS, and functional impairment, assessed by psychosocial measures. The strongest associations will be apparent for the combination of these markers.

MDD subtypes (Melancholia, Atypical MDD, Anxiety and Anhedonia), and the symptom classes of Pain (physical, psychological) and Sleep will be distinguished by the following candidate markers:

- Melancholia: Slowed information processing and slowed ERPs, greatest loss of grey matter/FA and reduction in brain activity. Associated with BDNF Met allele.
- Atypical: Variability of cognitive performance, EEG cortical and autonomic dysregulation, functional MRI connectivity and the greatest psychosocial impairment. Associated with MET variants.
- Anxiety: Greater exposure to early life stressors, higher neuroticism and negativity bias, and most impaired startle response and brain function during emotion tasks. Associated with 5HTT alleles.
- Anhedonia: Poorest general cognition and emotional intelligence, hypoarousal on autonomic and EEG measures and reduced neural synchrony. Associated with GABA variants.
- Pain will be associated with degree of performance/activity on emotion and startle tasks in particular and with autonomic arousal and activity in anterior cingulate.
- Sleep problems will be associated with poor general cognitive performance and with EEG alpha-slow wave alterations, reflecting thalamo-cortical arousal.

Pilot data. Using the same methodology as proposed for iSPOT-D, we have previously demonstrated that a number of these objective markers distinguish sub-clinical depression, consistent with the likely trait like status of the predicted markers (Kemp et al., 2005, 2006; Sumich et al., 2006; Williams et al., 2007). The role of early life stress in impacting cognition and brain structure has also been demonstrated (Cohen et al., 2006). Figure 1 shows an example path model for sub-clinical depression, in relation to BDNF Met allele, its interaction with early life stress and prediction of depression mood via impact on brain and autonomic arousal – which forms part of the framework for the above predictions concerning relationships between hypothesised markers. Figure 1 is just one exemplar of the potential for these complementary measures to act as markers of depression and treatment prediction.

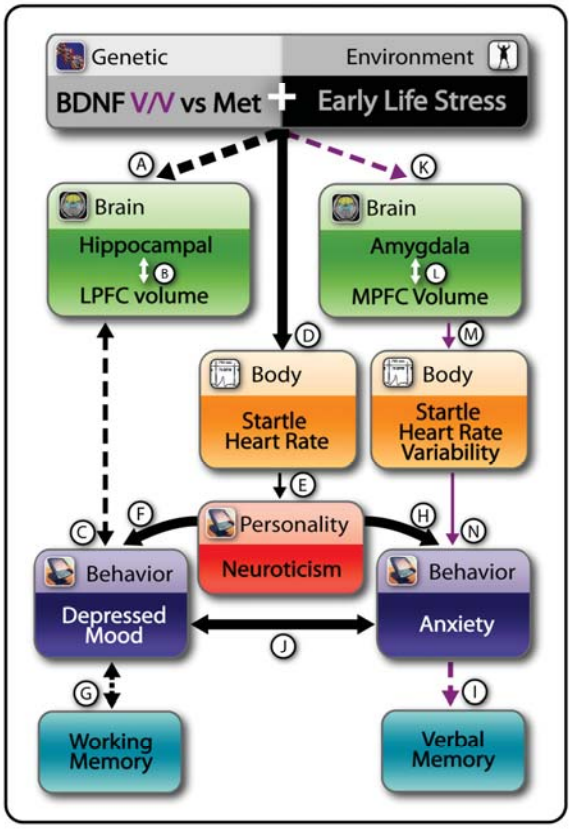

Solid lines represent positive path coefficients, dashed lines represent negative path coefficients, single-headed arrows represent direct effects, and double-headed arrows reflect correlation coefficients. The thickness of the line represents the significance of the effect (thin, medium, thick lines corresponding to p < .05, .01 .001, respectively). The interaction of BDNF Met status with exposure to ELS contributed to distinctive brain-arousal pathways to trait depression with comorbid anxiety (indicated by black lines). The interaction of BDNF Met status and ELS directly predicted reduced hippocampal volume (**A.** b=-0.11, p<.001). This reduction was correlated with reductions in lateral prefrontal cortical volume (**B.** r=0.68, p<.001), which in turn was associated with greater depressed mood (**C.** r=-0.09, p<.01). The interaction of BDNF-ELS predicted increases in startle-elicited heart rate (**D.** b=4.59, p<.001), which predicted increased neuroticism (**E.** b=0.19, p<.05), and subsequently increased trait depression (**F.** b=0.34, p<.001) and increased trait anxiety (**H.** b=0.11, p<.001). Trait depression was associated with poorer working memory accuracy (**G.** r=-0.75, p<.01), whereas higher anxiety predicted poorer performance on a distinct aspect of memory; verbal memory (**I.** b=-0.08, p<.01). Depression and anxiety were positively correlated (**J.** r=0.42, p<.001). In contrast, the interaction of BDNF V/V genotype with exposure to ELS predicted elevated trait anxiety via an alternative neural system (indicated by purple lines). Compared to Met carriers, the interaction of BDNF V/V status and ELS predicted enlarged amygdala volume (**K.** b=-0.03, p=.003), which correlated positively with medial prefrontal volume (**L.** r=0.65, p<.001). Enlarged amygdala-medial prefrontal volume predicted elevations in startle-elicited heart rate variability (**M.** b=3.53, p<.05), which was associated with elevated trait anxiety (**N.** b=0.08, p<.05).

Figure 1. Example path model from sub-clinical depression for the effects of BDNF Val66Met genotype and its interaction with early life stress (ELS) on brain, arousal and negative mood.

The primary hypotheses for markers of MDD treatment response will also focus on the most robust research evidence to date (eg. Bruder et al., 1999; Venn et al., 2005).

1. In regard to Objective 2, the following hypotheses concern the candidate markers which will change with treatment response (defined as a 50% reduction from baseline), following acute (8 weeks) drug treatment in MDD:

- General cognitive performance (with the exception of information processing speed), social cognition, EEG, arousal, and brain function assessed by fMRI will ‘normalise’ with SSRI, SNRI and NDRI drug treatments, defined by a significant shift in the direction of normal control performance.
- The degree of shift in the above measures will be associated with genotype and loss of grey matter/FA.
- The individual measures which shift with treatment will vary according to type of treatment, as will their associations with genotype and loss of grey matter/FA, with working hypotheses as follows:
    i. Social cognition: Improvements in emotion recognition will differ according to serotonergic and noradrenergic compounds (Venn et al., 2005).

ii. Electrical Brain Function: ERPs and neural synchrony to individual emotions will distinguish degree of response to serotonergic versus noradrenergic treatments.
iii. fMRI. Given associations between anterior (especially subgenual) cingulate with serotonin binding, it is also expected that reductions in anterior cingulate activity will show a greater improvement with SSRIs versus other drugs.
iv. Genetic: Specific variants of serotonin polymorphisms will have better responses to the SSRI Escitalopram (eg. Choi et al., 2005) while those with specific noradrenalin variants (such as -182C of NET) will have better responses to SNRI.

Primary hypotheses for markers of MDD treatment prediction will also focus on the research evidence to date. In regard to Objective 3, the following hypotheses concern the candidate markers which will predict

1. The following candidate markers will predict types of treatment response ('placebo', response, asymptomatic, remission, recovery).

- Poor response and lack of recovery will be predicted by the following combination of markers:
  i. Slowed information processing speed, particularly in the sensori-motor domain.
  ii. More pronounced EEG Alpha asymmetry (greater right than left activation) and dysregulated EEG power will be predictive of poorer treatment response.
  iii. Similarly, slowed and reduced ERPs during memory and attention tasks
  iv. Greatest loss of grey matter/FA.
  v. The interaction of BDNF Met allele and exposure to a high level of early life stress.
  vi. It will also be associated with the Melancholic sub-type in particular.

- Remission and recovery will be predicted by the reverse pattern of EEG asymmetry.

2. Brain measures (fMRI, EEG) will enhance the prediction of SSRI response to both clinical and behavioural measures, over and above the genetic contribution. For example, responders will be characterised by at least one copy of the L-allele of the 5-HTT and increased off-medication anterior cingulate activation, while non-responders will be characterised by at least one copy of the short allele of the 5-HTT and increased amygdala (as well as decreased anterior cingulate) activation during presentation of fearful facial expressions (Genotype X Neuroimaging interaction).

3. The following candidate markers will distinguish prediction of treatment response for different types of treatment:

- Social Cognition: Biases to recognition of individual emotions will show differential prediction of response/remission/recovery for serotonergic and noradrenergic compounds.

- Electrical brain function: ERPs and neural synchrony to individual emotions will show differential prediction of response/remission/recovery for serotonergic and noradrenergic treatments.

- Concurrent arousal: Alterations in autonomic arousal (and interaction with stress) will predict response/remission/recovery to noradrenergic agents in particular, given the noradrenergic modulation of the HPA axis (Hasler et al., 2004). By contrast, biases in startle modulation (eg. greater responses to negative emotion facilitated startle) will predict responses to serotonergic agents, given evidence for a serotonergic role in such modulation.

- Genetic: MDD subjects with specific variants of serotonin polymorphisms (such as -1438A/G of 5HT2A) will be more likely to remit and recover following SSRI Escitalopram (eg Choi et al., 2005) while those with specific noradrenalin variants (such as -182C of NET) are more likely to remit and recover following venlafaxine.

4. It is expected that the above markers will predict degrees of remission and recovery, defined according to both symptom improvements and psychosocial functioning improvements.

Primary hypotheses for identifying individual characteristics which predict response to treatment in MDD

Multivariate analyses such as discriminant function analysis will be used to determine if specific subtypes of MDD, and associated cognition-brain-gene clusters of individual's distinct differential responses to acute treatment.

1. It is expected that individual types of MDD can be identified in terms of both clinical and cognitive-biological characteristics that predict responses to treatment and to a particular type of treatment.

10.2.2 Secondary Hypothesis

A secondary set of hypotheses will be tested in relation to the secondary questions addressed in this trial, including:

1. The prediction that the markers of MDD and sub-types identified in the primary analyses will also distinguish clusters of co-morbid conditions in MDD.

2. That the extent of change in markers which change with treatment will be moderated by other subject characteristics such as age and sex.

3. That markers predicting treatment response identified in primary analyses will also predict other aspects of drug response, such as number of side effects.

10.3 Sample Size and Power

The large number of subjects (n=2,688) is targeted to ensure a high level of statistical power to evaluate the multiple objective markers in the study. The alpha level will be set at a corrected threshold of .008 (with a family-wise correction for multiple markers; n=165) and use the mean difference (change in pre-post treatment) scores and standard deviations for the mean of population 1. Assuming a minimum effect size of .3SD relative to the current standardized control-database, power was calculated for detecting a difference in treatment effect across Control versus MDD treatment groups. Power calculations were performed using Russ Lenth's online power calculators (http://www.stat.uiowa.edu/~rlenth/Power/index.html). Using an effect size of 0.3 (assuming 50% more variance in the clinical group) to achieve a statistical power of at least .80 requires groups of n=672 (including allowance for drop-out). This effectively ensures a minimum power of .80 for any comparisons of the difference *between* treatment groups. This figure ensures that sufficient power is also still attained for sub-group analyses of moderate versus high severity of symptoms, and is sufficient to deal with unequal sized groups.

In addition to traditional significance testing, verification of findings in independent groups is always desirable. In this case, we target the full n=672 to investigate markers of the three different treatments in MDD. However, this allows us a total group of 672 x 3 (2,016) to investigate markers of MDD. Given the large sample size and the full range of biological measures, we will undertake a split of the sample to confirm findings concerning the objective markers, at the end of the trial. Candidate markers will be identified in the first half of the study and potentially confirmed in the second half of the study (as proposed by the FDA).

10.4 Data Reduction

Data reduction and scoring of raw EEG data and touchscreen data will occur at the centralized analysis facility.

Psychological test markers

The measure of exposure to early life stress is quantified in terms of number of stressful events, and this number may be further classified in terms of the age band within which the exposure occurred.

Cognitive test markers

Scores are performance scores in each test: including accuracy, reaction time and total number values. Factor scores will also be derived for the composite domains.

Electrical Brain Function markers
EEG

EEG Power: For each average power spectra, the power will be calculated in the four frequency bands, delta (1.5 - 3.5 Hz), theta (4 - 7.5 Hz), alpha (8 - 13 Hz), and beta (14.5 - 30 Hz). This power data will then be square-root transformed in order that it might better approximate the normal distributional assumptions required by parametric statistical methods.

EEG Asymmetry: Calculation of asymmetry scores for frontal (eg. F7-F8 and F3-F4) site pairs, and any required controls (eg. Parietal asymmetry) for the Alpha band.

EEG synchrony: Quantification of phase locking which is independent of power, particularly for the high frequency (Gamma, 39-41Hz) band, but also for the traditional Alpha, Beta, Theta, Delta bands.

ERPs

ERPs are scores for the components of key interest in each task. For instance:

Auditory Oddball:
- N100 (negative deflection 100 ms post-stimulus), indexing initial attention.
- P200 (positive deflection 200 ms post-stimulus), decision-making.
- P300 (positive deflection 300 ms post-stimulus), context evaluation and updating.

Working Memory
- P450 (positive deflection 450 ms post-stimulus), working memory updating.

Go NoGo
- N200 (negative deflection 200 ms post-stimulus), inhibition for NoGo stimuli, frontally.
- P300 (positive deflection 300 ms post-stimulus), as above.

Facial Emotion
- P200 (or VPP), which is modulated by emotion, and reduced in depression frontally.
- P300 (positive deflection 300 ms post-stimulus), controlled evaluation of emotion.

Heart Rate
Heart rate (beats per minute) and Heart rate variability will be quantified

Skin Conductance
The sweat rate will be decomposed into a tonic (skin conductance level [SCL]) component as a function of time, and a phasic (skin conductance response [SCR]) component. Multiple, overlapping SCRs will be separated by estimating the time-course of the sudomotor nerve activity underlying the electrodermal time-series, and scored for onset-time, rise-time, peak amplitude and decay half-height.

EMG Startle
EMG responses to each startle/prepulse stimulus, that rate of habituation and difference between startle and prepulse stimuli will be calculated.

Structural MRI
Analysis of the gray, white and CSF distribution and overall volume in T1-weighted images will be performed in the SPM package using voxel-based morphometry (VBM). The processing protocol used in VBM have been published using a brain resource-specific template (Grieve et al., 2005), based on those of Ashburner & Friston (2000) and Good et al. (2001). Briefly, the brains are first spatially normalised by transforming each brain onto the Standardised template which approximates Talairach space. The pixels within each brain volume are then segmented into gray, white, CSF and non-brain portions based on a cluster analysis method to separate pixels based on intensity differences, and a priori knowledge of known tissue distributions in normal individuals. A correction is also made for

image non-uniformity. Following tissue segmentation an intensity correction is made based on the deformation field used in the initial spatial normalisation process, this correction adjusts for the distortions of normalization and makes the measured volumes absolute (Ashburner & Friston, 2000).

We are also able to apply an ROI approach, in which grey matter volumes are extracted for 106 brain regions according to the AAL atlas.

DTI analysis based upon predictions of network dysfunctions, will also be undertaken.

Functional MRI
Data are also processed using SPM on a Matlab platform, using comparisons which correspond to those for ERPs. Time series of BOLD signal change can be exported for the same 106 brain regions articulated in the AAL neurological atlas.

Genetics
Genetic data analysis involves the extraction of genotype information from DNA samples. Initial analysis will focus on candidate polymorphisms for major depression and for predicting treatment response in depression, which include those with some existing support from published studies (eg. Antilla et al., 2007; Caspi et al., 2003; Choi et al., 2005 ; Gatt et al., 2007a,b ; Kim et al., 2000 ; Lewis et al., 2006; Lemonde et al., 2003; Oruc et al., 1997 ; Ryu et al., 2006 ; Zhang et al., 2005).

A selected list of initial targets is provided below:

- SERTPR (5HTT short allele), risk for depression, particularly in females and interaction with stress and prediction of response to SSRIs (Caspi et al., 2003; Kim et al., 2000).
- BDNF Val66Met (Met allele).
- C1019 G (5HT1A) (in regard to risk for MDD, and effect of SSRIs. Interaction with BDNF Met predicts treatment-resistant MDD; Antilla et al., 2007).
- 182C NET gene (in regard to risk for MDD and effect of SNRIs and NRIs; Ryu et al., 2004).
- 1438A/G and 102 T-C (5HT2A) in regard to risk for MDD and better response to Citalopram (Choi et al., 2005).
- BCL-2 in regard to serotonergic treatment for MDD (Chen et al., 2007).
- Trytophan hydroxylase-2 TPH2 (G1463A) in regard to impact of stress, serotonergic system and treatment-resistant MDD.
- Chromosome region 15q14 (eg. GABRA5) in regard to MDD, and mechanisms of treatment (Oruc et al., 1997).

We have shown the enhanced statistical power and functional significance of adding cognitive and brain measures. For example, BDNF Met carriers are at higher risk of depression via impacts on these measures (Gatt et al., 2007a,b).

Array analyses will be used to extract additional significant genotypes, not yet associated with these disease groups in the published literature, as exploratory candidate markers.

Analysis will yield a genotype grouping for each subject, which can be expressed as number of alleles (allele loading). Subjects can therefore be coded according to number of a particular allele, as 0, 1 or 2 (corresponding to 2, 1 or 0 of another allele respectively).

The frequency of genotypes will first be validated for each group of subjects using the Hardy-Weinberg principle to ensure that the distribution of genotypes accords to that expected for the general population.

This principle is summarised in Figure 2. Satisfying this condition reduces the incidence of type I error, and allows confidence in the results of the genetic analysis (e.g., Salanti et al, 2005).

Allele loading will be included as a covariate of interest in the planned analyses, providing information on whether genotype is an important mediator contributing to Disease Markers and Treatment Markers. For instance, a significant positive covariation between COMT allele loading and a candidate Disease Marker would indicate that the Marker is even more extreme in subjects with a higher loading of that allele.

Hardy-Weinberg principle

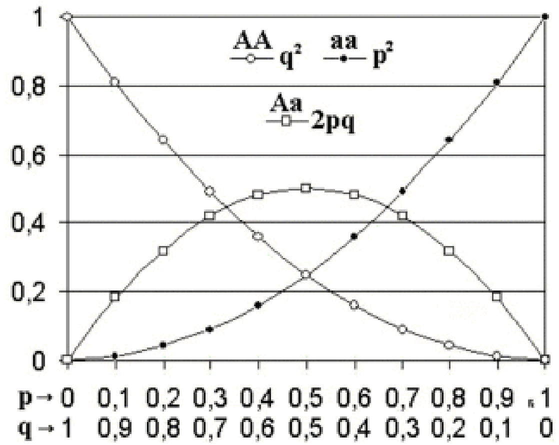

Hardy–Weinberg principle for two alleles: the horizontal axis shows the two allele frequencies p and q, the vertical axis shows the genotype frequencies and the three possible genotypes are represented by the different glyphs.

In population genetics, the Hardy–Weinberg principle (HWP) states that, under certain conditions, after one generation of random mating, the genotype frequencies at a single gene locus will become fixed at a particular equilibrium value. It also specifies that those equilibrium frequencies can be represented as a simple function of the allele frequencies at that locus.

In the simplest case of a single locus with two alleles A and a with allele frequencies of p and q, respectively, the HWP predicts that the genotypic frequencies for the AA homozygote to be p2, the Aa heterozygote to be 2pq and the other a homozygote to be q2. The Hardy–Weinberg principle is an expression of the notion of a population in "genetic equilibrium" and is a basic principle of population genetics.

Figure 2. Summary of the Hardy-Weinberg equilibrium principle.

10.5 Planned Analyses

Planned analyses address the goal of identifying Disease Markers of Depression (compared to normative controls) and Treatment Markers of change with medication, and which predict response to treatment. A summary of cell numbers if provided in Table 1.

Table 1. Summary of cell numbers for between and within-group analyses:

Subject Group	Treatment Group*	Baseline (n)	Post-medication (n)	TOTAL observations
MDD	Escitalopram, Sertraline Venlafaxine XR	2,016 (672x3)	2,016 (672x3)	4,028
Controls	N/A	672 (672x1)	672 (672x1)	1,344
				5,372

* Randomised allocation

In the planned analysis, dependent measures are the scores from the genetic, cognitive, electrical brain function (EEG, ERP) and MRI/fMRI batteries. These are referred to as ‘cognition-brain’ markers. Two sets of analyses will be undertaken. The focal set will be undertaken with all subjects (ie. with the subject numbers indicated below). The second set will be undertaken for the subset of subjects with MRI markers in addition to all other measures. Key screening and genetic data (in allele loading) may be included as covariates of interest, given they are pre-existing factors.

In addition to multivariate analyses of clinical differences and treatment response prediction, univariate analyses of each of the 165 key measures[†] will be performed.

The goal of the univariate analyses is to ‘database’ the profile of differences between depressed and healthy subjects, and differences between off and on-medication profiles for people with MDD. This trial will present a unique opportunity to obtain values approaching ‘true scores’ for the populations in question, due to the large sample size employed in the study (n=672) and the large sample size of the reference database (BRID, N>4,000). With increasing sample size, the discrepancy between the sample mean and the population mean decreases asymptotically (see figure 3). This means it is statistically very unlikely that average scores for a large sample differ much from ‘true’ scores in the general population. Furthermore, the increased sample size, and statistical power allows a robust correction for multiple comparisons to be made to minimise false positives in the profile obtained.

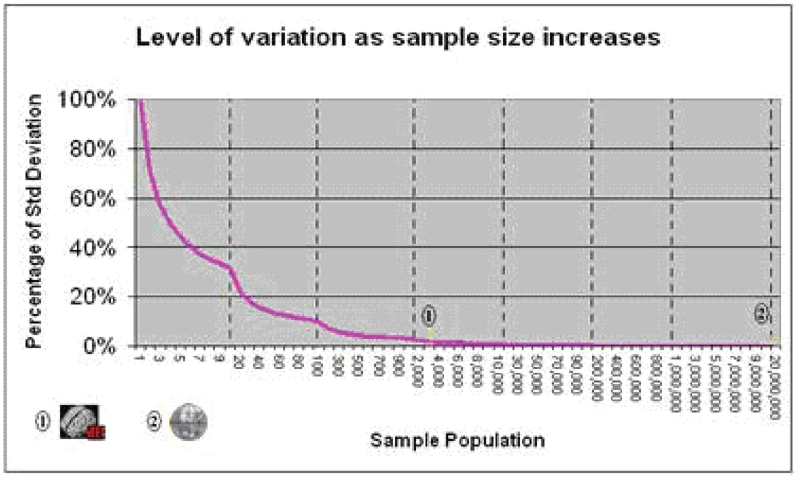

Figure 3: The phenomenon of ‘regression to the mean’ (Galton, 1886), entails that as sample size increases, the likelihood of a sample differing statistically from the general population decreases.

[†] Note that some of these 165 measures are composite measures derived through various data reduction techniques.

Between groups

1. (a) Univariate Analysis Determine Markers which significantly distinguish MDD from controls.

DEPRESSION: 2016 MDD versus 672 control one way ANOVAs for cognition-brain markers (DVs) to establish profile markers of Depression.

DEPRESSION: 2016 MDD versus 672 controls using repeated measures ANOVAs for cognition-brain markers (DVs) which form within-subjects repeated measures factors (eg. or multiple brain regions for EEG and ERPs).

Genotype (allele loading) and personal history factors (such as exposure to trauma) may be included as covariates of interest.

(b) *Determine which combination of markers provide the best profile of MDD Markers.*

DEPRESSION: 2016 clinical versus 672 control multivariate analyses (such as logistic regression) to determine which combination of measures provides the best profile Marker discrimination of Depression from controls.

Genetic allele loading may be included as a prediction variable of interest.

(c) Testing the assumption of random drug allocation. To establish equivalence of randomly allocated drug groups, test that whole MDD group baseline differences to controls are the same across treatment sub-groups.

DEPRESSION: similar analysis to (a) above, but with the depression group split into three, using contrasts: 1, -1, 0, 0; 1, 0, -1, 0; 1, 0, 0, -1; 0, 2, -1, -1; 0, 0, 1, -1. [where groups are controls, drug-E, drug-V, drug-B].

One commonly problematic feature of clinical trials using treatment prediction is the criteria for identifying successful treatment. Typically, a symptom scoring instrument (such as the HAM-D for MDD) is used with a critical threshold to evaluate response to medication. However, clinical 'signs and symptoms' are increasingly criticised for their subjectivity, and are not necessarily the best markers of treatment response (e.g., Moran, 2006).

A primary aim of this study is to establish objective and reliable biological markers for diagnosis and treatment evaluation. To this aim, treatment response will not simply be measured by change in clinical rating (e.g., HAM-D), but also through normalisation (change from outside to within normal range) of baseline differences in markers. As a complement to previous research using clinical symptoms, markers that improve concurrently with clinical symptoms will be identified through correlation analyses.

Off vs On-Medication

2. (a) To determine Treatment Markers which identify which markers [from (1)] improve/normalise with medication.

For each drug the within the Depression group:

One way ANOVAs with difference scores on-medication, minus off-medication across clinical-versus-control groups (can be also formulated as a contrast, or a t-test, as it is a 1df test) for each DV.

Repeated measures ANOVAs for cognition-brain markers that form within-subjects repeated measures factors (eg. or multiple brain regions for EEG and ERPs).

Genotype (allele loading) and personal history factors (such as exposure to trauma) may be included as covariates of interest (ANCOVA).

(b) *Determine which combination of markers provide the best profile of Treatment Markers*.*

For each drug the within the Depression group:

Multivariate correlation analyses (such as regression analyses) to determine which combination of markers provides the best prediction of change post-medication with each compound.

(c) Aim to identify which objective Treatment Markers relate to subjective clinical ratings of improvement/normalisation (using coordinator ratings HAM-D[21]*, QIDS-SR depression rating scale) and functional outcome (using quality of life, SF-12 and other measures of social function).*

Correlate changes in pre-post medication performance (difference scores) with difference scores from subjective clinical ratings and functional outcome measures. Correlations provide validity for medication response profile against these subjective ratings.

3. To determine if Treatment Markers predict level of response to compound by grouping subjects into responders and non-responders based on a 50% reduction of HAM-D scores. Logistic regression analyses where response/non-response is the dependent variable and potential markers are test iteratively to find an optimal combination. Selected combinations will be validated using a second dataset for confirmation.

4. To determine if Treatment Markers predict level of response [based on marker normalisation with treatment] to compound by grouping subjects into high responders, average responders, non-responders, and any who get worse with treatment, on these markers.

Regression analyses with two subject groupings assigned by the following:

i. Allocate each subject a score based on a regression-style equation weighting on the most robust measures of treatment response for the group as identified in 1(c) above.
ii. Using an individualised approach, in which evaluation of response is based on improvement on measures showing the greatest deficit pre-treatment for each individual subject (i.e., using level of remission according to the QIDS-SR).

Between Compounds

5. In (2), determine if compounds differ in the degree of improvement/normalisation on the Treatment Marker profile.

Examine descriptives for the profile of response across Treatment Markers. Test these differences using ANOVAs with Dunnett's test for each drug group versus the control group.

6. In (4), examine any differences in proportions of high/average/no/negative response across compounds within MDD.

Test for differences between compounds in patterns of treatment response using CHI SQUARE.

Exploratory Analyses / Datamining

7. Exploratory: Analysis to determine markers for optimal treatment prediction accuracy within each compound using datamining techniques.

Exploratory analyses and datamining will be performed at the interim analysis stage after the first half of data has been acquired. All potential markers will be re-examined in the final analysis to ensure that they are replicated in the data from the second half of the study.

These analyses are likely to involve exploratory data techniques such as permutation analysis and decision tree methods which are particularly useful for combining data from different domains (e.g., Goldman et al, 1982).

Markers could come from any one or combination of the domains, screening, genetics, general and social cognition, EEG, ERPs, fMRI, sMRI, autonomic arousal. In addition to multivariate techniques (such as logistic regression), we will also use hypothesis-driven approaches such as path modelling (structural equation modelling).

Interim Analysis:

> An interim analysis will be performed when the first half of the data (subjects completed in each drug condition > 335) has been collected. All protocol analyses listed above will be performed. Upon collection of the full dataset, in addition to the main analysis using the full sample, confirmation of findings from the interim analysis using only the second half of the dataset will be sought.

Treatment of missing data, skewed data, and outliers:

> In the likely event of missing data, data will be imputed if deemed appropriate using procedures dependent on the extent and type of missing data (e.g., Bayesian or regression methods). Excessive amounts of missing data for a given measure (>15% of cases), or a given individual (>15% of subject dataset) will be left as missing.
>
> Log transformation will be used on appropriate variables to achieve an acceptably normal distribution where tests/models require it. Based on the existing Brain Resource International Database, distributions of most measures being collected are known, and transformations will be performed according to those established in current Brain Resource methodology manuals.
>
> Outliers will be treated either by removal or windsorization methods. Outlier *removal* will be preferred where there is evidence that outliers reflect a qualitatively different measurement to the expected distribution, e.g., artefact, equipment error, misunderstanding of test instructions, etc. Removed outliers may be replaced with an imputed value based on the same criteria for missing value replacement. For outliers where scores fall within a range likely to reflect a genuine measurement - although an extreme one - windsorization replacement techniques will be preferred (outliers are scaled toward the mean, but the order of scores is preserved).

Individual difference (Personalised) analyses

Measures will be normalised using a Non-Linear Regression Model; a ‘peer referencing’ approach. This model is based upon John Crawford’s (Aberdeen University) model and his normalization procedure (Crawford and Howell, 1998). The model is built using data from a large number of census matched norms – a control group especially selected for data integrity, normality and conformity to Census demographic distributions. It enables use of the entire database as controls, rather than a small matched sample, enabling greater confidence in the normative score. The model has a high utility as it explains up to 60% of the variance for variables examined to date.

The quadratic trends observed for general cognitive performance (across tests) and EEG Theta power (see figure 4) provide typical examples of the trends seen for the normative Brain Resource International Database. In these examples, note that with the steady age-decline, that the difference between the average 50 year old and the average 65 year old is around 1 full standard deviation [scale is Standardised to mean = 100, standard deviation = 15].

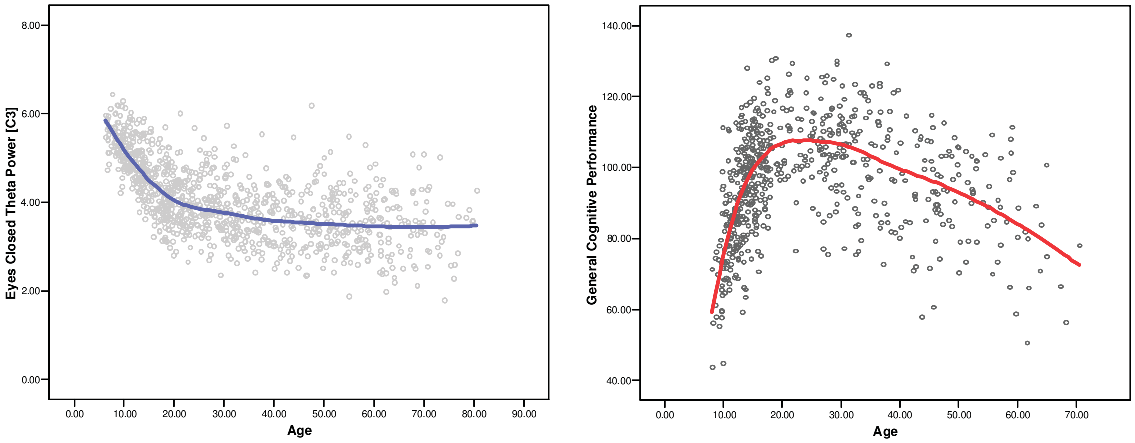

Figure 4. Examples of EEG power (left) and general cognitive performance (right) scores over age. Variance due to age, or any other demographic variable of interest in this study (individually or as a combination of variables) can be taken into account using a peer-referencing technique which normalises for the variance due to these variables.

The peer-referencing technique normalises not only the effects of age, but also sex and education level – or other demographic and history measures of interest (see figure 5). This method places individual scores in a clear context of performance, relative to their peers. This peer-referencing technique facilitates personalised analyses of individual cognitive and brain function profiles.

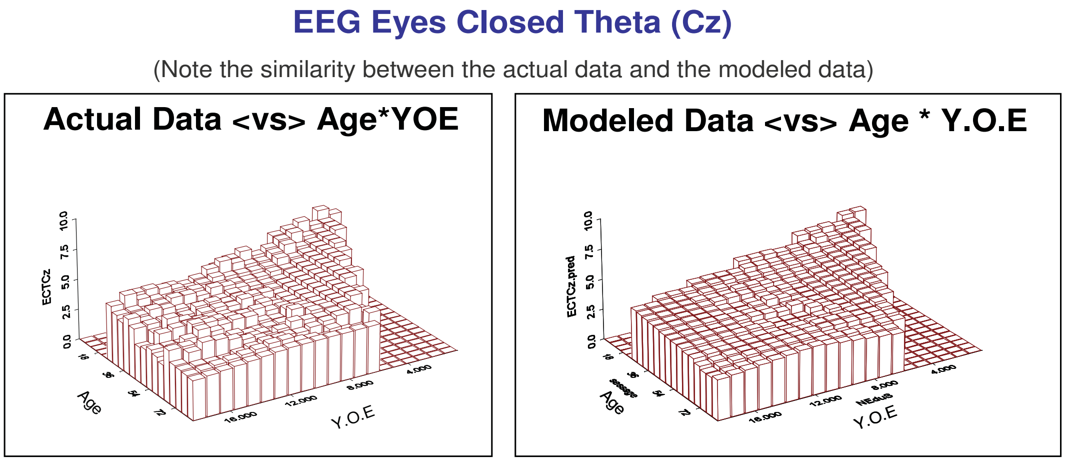

Figure 5: Peer regression modelling is not restricted to age trends, but can also account for additional dimensions such as education level (Y.O.E = years of education above). It can be seen from the small magnitude in difference between the actual data and modelled data that much of the variance of EEG scores (for example) can be accounted for by age and gender. By adjusting for this proportion of the variance due to demographic factors, individual differences are highlighted provided enhanced statistical power, particularly for personalised analyses.

This sort of analysis allows trial data to be placed in context of existing database scores (for all measures in this study). Trial control data can be compared to database controls to demonstrate the representativeness of the recruited control group. Furthermore, interactions across age are easily identified, for instance, if any markers of depression decline faster with age, than for controls – over and above a mean difference – then this will be testable using peer regression modelling (see figure 6).

Using peer-referenced scores, the profiles of individual MDD subjects on cognitive and brain measures, and in relation to genetic variants, may be identified.

Similarly, individual profiles of <u>every</u> cognitive and brain function predictors of treatment (as well as in relation to genetic variants), can be examined in this manner.

Bayesian methods can be used with these peer-referenced scores to quantify the probability of a successful treatment response for a given individual, based on trends within the relevant group.

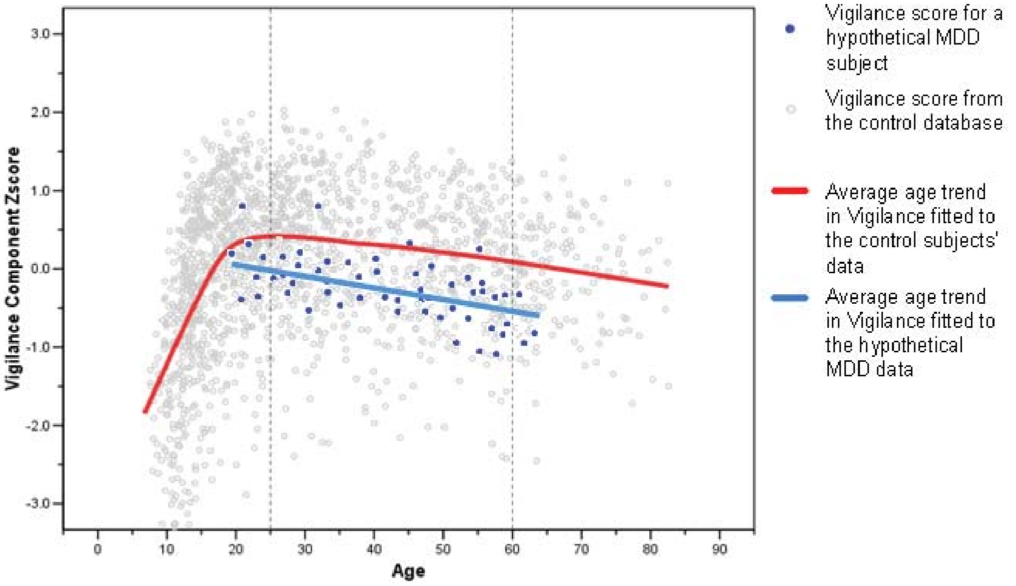

Figure 6: The graph above shows how the MDD data may look in comparison to controls from the existing Brain Resource International Database. We might predict that MDD subjects would have lower levels of vigilance than the general population, but levels of vigilance would vary between individuals and would generally show a subtle decline with age, and perhaps a steeper decline than controls due to cumulative effects of depression over the lifespan.

As illustrated below, the database will be used to elucidate the extent of change in individuals, with each medication used.

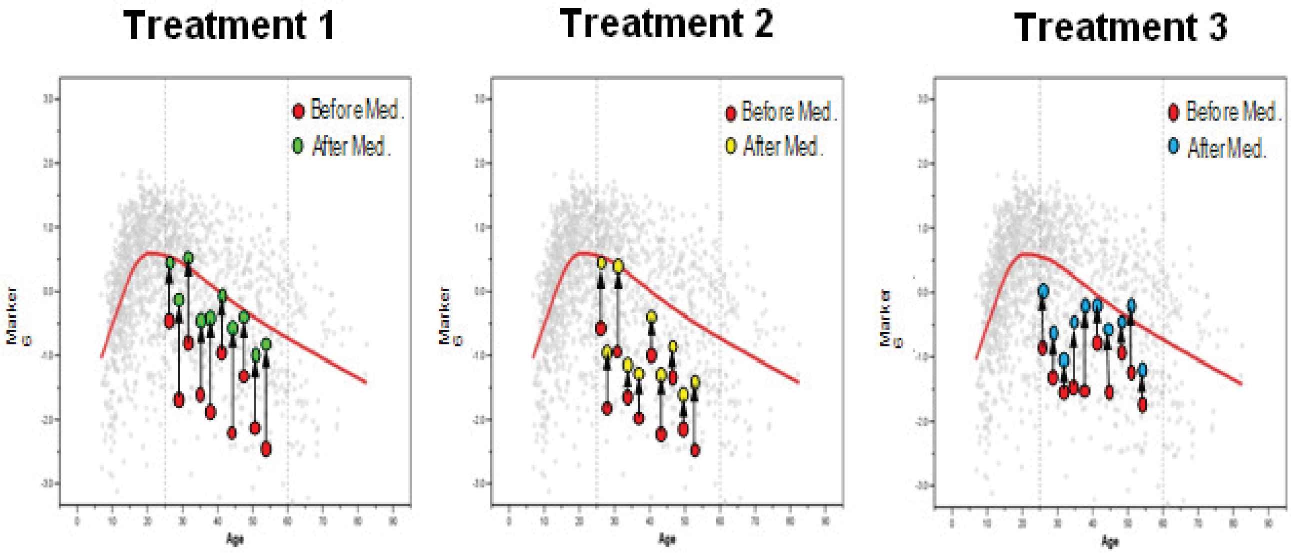

Figure 7: Individualized treatment response to show best relative responses and the <u>extent</u> of individualized response, using the database norms as a frame of reference.

In essence, the confluence of standardization, integration and scale in this study, is likely to adequately test the hypotheses concerning MDD markers and personalised prediction of treatment response.

11. REFERENCES

Anttila S, Huuhka K, Huuhka M, Rontu R, Hurme M, Leinonen E, Lehtimaki T. (2007). *J Neural Transm.*(March, online ahead of publication).

Ashburner J & Friston KL (2000). Voxel-based morphometry – the methods. *Neuroimage* 11: 805-821.

Bruder, G.E., Tenke, C.E., Stewart, J.W., McGrath, P.J. and Quitkin, F.M. (1999). Predictors of therapeutic response to treatments for depression: A review of electrophysiologic and dichotic listening studies. *CNS Spectrums* 4, pp. 30–36.

Caspi, A., Sugden, K., Moffitt, T.E., Taylor, A., Craig, I.W., Harrington, H.L., McClay, J., Mill, J., Martin, J., Braithwaite, A and Poulton, R (2003). Influence of Life Stress on Depression: Moderation by a Polymorphism in the 5-HTT Gene. *Science,* 301 (5631): 386-389.

Chen, S. J., Kao, CL., Chang, Y.L., Yen, C.J., Shui, J. W., Chien, C.S., Chen, I. L., Tsai, T. H., Ku, H. H., & Chiou, S. H. (2007) Antidepressamt AdMINI Plusstration modulates Neural Stem Cell Survival and Serotoninergic Differentiation Through bcl-2. *Curr Neurovasc Res 4(1): 19-29.*

Choi M-J, Kang, R-H, Ham B-J et al. (2005). Serotonin Receptor 2A Gene Polymorphism (-1438A/G) and Short-Term Treatment Response to Citalopram. *Neuropsychobiology*;52:155-162.

Clark, C. R., Paul, R.H., Willimas, L.M., Fallahpour, K., Handmer, C and Gordon, E (2006). Standardised assessment of cognitive functioning during development and aging using an automated touchscreen battery, *Archives of Clinical Neuropsychology,* 21: 449-467.

Clark, L. A., & Watson, D. Tripartite model of anxiety and depression: Psychometric evidence and taxonomic implications. Journal of Abnormal Psychology. 1991;100:316-336.

Cohen, R.A., Grieve, S., Hoth, K., Paul, R.A, Sweat, L., Tate, D., Gunstad, J., Stroud, L., McCaffrey, J., Hitsman, B., Niaura, R., Clark, R., MacFarlane, A., Bryant, R.A., Gordon, E & Williams, L.M (2006). Early life stress and morphometry of the adult anterior cingulate cortex and caudate nuclei, *Biological Psychiatry*, 59: 975 - 982.

Crawford, J.R. and Howell, D.C. (1998). Regression Equations in Clinical Neuropsychology: An Evaluation of Statistical Methods for Computing Predicted and Obtained Scores. *Journal of Clinical and Experimental Neuropsychology*, 20(5): 755-762.

Gatt, J.M., Clark, C.R., Kemp, A.H., Liddell, B.J., Dobson-Stone, C., Kuan, S., et al. (2007) A Genotype-Endophenotype-Phenotype Path Model of Depressed Mood: Integrating Cognitive and Emotional Markers, *Journal of Integrative Neuroscience* 6(1): 75–104.

Gatt, J.M., Kuan S, Dobson-Stone, C., Paul, RH, Joffe RT, Kemp A, Gordon E, Schofield PR, Williams LM (2007b). Alpha band neural activity mediates the role of the BDNF Val66Met polymorphism in risk for depressive features. *Neuropsychopharmacology* (In Press).

Goldman L, Weinberg M, Weisberg M, et al *(1982).* "A computer-derived protocol to aid in the diagnosis of emergency room subjects with acute chest pain". N. Engl. J. Med. 307 (10): 588-96

Good CD, Johnsrude IS, Ashburner J, Henson RN, Friston KJ, Frackowiak RS (2001). A voxel-based morphometric study of ageing in 465 normal adult human brains. Neuroimage 14: 21-36.

Gordon, E. (2007). Genomics and Neuromarkers are both required for the era of brain-related 'Personalised Medicine". *Personalised Medicine* *4(*2):201-215.

Gordon, E., Cooper, N., Rennie, .C, Hermens, .D and Williams, L.M (2005). Integrative neuroscience: the role of a standardised database. Clinical EEG and Neuroscience, 36: 64 - 75.

Gratton, G., Coles MG & Donchin E (1983). A new method for off-line removal of ocular artifact. Electroencephalography and Clinical Neurophysiology, 55: 468-484.

Grieve, S., Clark, C.R, Williams, L.M and Gordon, E (2005). Preservationn of limbic and paralimbic regions with aging. *Human Brain Mapping*, 25: 391-401.

Hasler G, Drevets WC, Manji HK, Charney DS. (2004). Discovering Endophenotypes for Major Depression. Neuropsychopharmacology, 29, 1765–1781.

Iannuzzo RW, Jaeger J, Goldberg JF, Kafantaris V, Sublette ME. (2006). Development and reliability of the HAM-D/MADRS interview: an integrated depression symptom rating scale. Psychiatry Res. 145, 21-37.

Insell, T.R. (2006). Beyond efficacy: The STAR*D trial. Am. J Psychiatry, 163: 5-7.

Keller MB. (2003). Past, present, and future directions for defining optimal treatment outcome in depression: remission and beyond. JAMA. 289, 3152-60.

Kemp, A.H., Stephan, B.C.M., Hopkinson, P., Sumich, A.L., Paul, R.H., Clark, R.C., Bryant, R.A., Gordon, E and Williams, L.M (2005). Toward an Integrated Profile of Depression: Evidence from the Brain Resource International Database. Journal of Integrative Neuroscience, 4: 95 - 106.

Kemp, A.H., Cooper, N.J., Hermans, G., Gordon, E., Bryant, R and Williams, L.M. (2005b). Toward an integrated profile of emotional intelligence: Introducing a brief measure. *Journal of Integrative Neuroscience,* 4(1): 41-61.

Kemp, A.H., Hopkinson, P.J., Stephan, B.C.M., Clark, C.R., Gordon, E., Bryant, R and Williams, L.M (2006). Predicting Severity in Non-clinical Depression: Preliminary Findings using an Integrated Approach. Journal of Integrative Neuroscience, 5: 89 - 110.

Kessler RC, Berglund P, Demler O, Jin R, Koretz D, Merikangas KR, Rush AJ, Walters EE, Wang PS (2003). The epidemiology of major depressive disorder: results from the National Comorbidity Survey Replication (NCS-R). National Comorbidity Survey Replication. 289:3095-105.

Kim, DK, Lim S-W, Lee S, Sohn SE, Kim Seonwoo, Hahn CG, Carroll B. (2000). Serotonin transporter gene polymorphism and antidepressant response. NeuroReport 11: 215-219.

Lavori PW, Rush AJ, Wisniewski SR, Alpert J, Fava M, Kupler DJ, Nierenberg A, Quitkin M, Sackheim HA, Thase ME, Trivedi M. (2001). Techniques and Methods Strengthening Clinical Effectiveness Trials: Equipoise-Stratified Randomization. *Biological Psychiatry*, 50: 792-801.

Lemonde, S., Turecki, G., Bakish, D., Du, L., Hrdina, P.D., Bown, C.D., Sequeira, A., Kushwaha, N., Morris, S.J., Basak, A., Ou, X.M and Albert, P.R (2003). Impaired Repression at a 5-Hydroxytryptamine 1A Receptor Gene Polymorphism Associated with Major Depression and Suicide. *Journal of Neuroscience,* 23: 8788 – 8799.

Lenth, R. V. (2006). Java Applets for Power and Sample Sise [Computer software]. Retrieved 5th January 2007, from http://www.stat.uiowa.edu/~rlenth/Power.

Lewis, S.J., Lawlor, D.A., Smith, G.D., Araya, R., Timpson, N., Day, I.N.M and Ebrahim, S (2006). The thermolabile variant of MTHFR is associated with depression in the British Women's Heart and Health Study and a meta-analysis. *Molecular Psychiatry*, 11: 352-360.

Lovibond, P.F., Lovibond SH. (1995). *Manual for the Depression Anxiety Stress Scales*, Psychology Foundation, Sydney, NSW.

Maislin, G., Pack, A.I., Kribbs, N.B., Schwartz, P.L., Schwartz, A.R., Kline, L.R., Schwab, R.J., Dinges, D.F. (1995). A Survey Screen for Prediction of Apnea. Sleep. 18, 158-166.

March JS, Silva SG, Compton S, Shaprio M, Califf R, Krishman R. (2005). The case for practical clinical trials in psychiatry. American Journal of Psychiatry, 162: 836-846.

Moran M (2006). Researchers Seek Drug That Targets Cognition. *Psychiatric News*, 41(2), 16.

Oruc L, Verheyen GR, Furac I, Ivezic S, Jakovljevic M, Raeymaekers P, Van Broeckhoven C. (1997). Positive association between the GABRA5 gene and unipolar recurrent major depression. Neuropsychobiology. 36(2):62-4.

Parker G., Hadzi-Pavlovic, D., (1996). "Melancholia: A Disorder of Movement and Mood", Cambridge University Press,. [Rating Scale Available at: http://www.blackdoginstitute.org.au/research/tools/documents/COREbooklet.pdf]

Paul, R.H., Gunstead, J., Cooper, N., Williams, L.M., Clark, R.C., Cohen, R.A., Lawrence, J.J and Gordon, E (2007). Cross-cultural assessment of neuropsychological performance and electrical brain function measures: Additional validation of an international brain database. *International Journal of Neuroscience,* 117:1-20.

Paul, R.H., Lawrence J., Williams, L.M., Clark, R.C., Cooper, N and Gordon, E (2005). The Validity of Integneuro: A new computerised and Standardised battery of neurocognitive tests. *International Journal of Neuroscience*, 115: 1549 - 1567.

Rogers, M.A., Bradshaw, J.L., Pantelis, C., Phillips, J.G. (1998). Frontostriatal deficits in unipolar major depression. *Brain Res Bull*, 47:297-310.

Rowe, D.L., Cooper, N.J., Liddell, B.J., Clark, R. C, Gordon, E. (2007). Brain Structure and Function Correlates of General and Social Cognition. *Journal of Integrative Neuroscience 6(1)*: 35-74.

Rush, A.J., Trivedi, M.H., Ibrahim, H.M., Carmody, T.J., Arnow, B., Klein, D.N., et al. (2003). The 16-item Quick Inventory of Depressive Symptomatology (QIDS) Clinician Rating (QIDS-C) and Self-Report (QIDS-SR): A psychometric evaluation in subjects with chronic major depression. Biological Psychiatry, 54, 573-583.

Rush A.J., Trivedi MH, Wisniewski SR, Nierenberg AA, Stewart JW, Warden D, Niederehe G., Thase ME, Lavori PW, Lebowitz BD, McGrath PJ, Rosenbaum JF. (2006). Acute and longer-term outcomes in depressed subjects requiring one or several treatment steps: A STAR*D report. *Am. J Psychiatry*, 163: 1905-1917.

Rush AJ, Fava M, Wisniewski SR, et al. (2004). Sequenced Treatment Alternatives to Relieve Depression (STAR*D): rationale and design. Control Clin Trials, 25, 119-42.

Ryu SH, Lee SH, Lee HJ et al. (2004). Association between norepinephrine transporter gene polymorphism and major depression. *Neuropsychobiology.* 49:174-7.

Salanti,G., Amountza, G., Ntzani, E.E., and Loannidis, J.P.A. (2005). Hardy–Weinberg equilibrium in genetic association studies: an empirical evaluation of reporting, deviations, and power. *European Journal of Human Genetics*, 13: 840-848.

Shear MK, Vander Bilt J, Rucci P, Endicott J, Lydiard B, Otto MW, et al. Reliability and validity of a structured interview guide for the Hamilton Anxiety Rating Scale (SIGH-A). Depress Anxiety. 2001;13(4):166-78.

Sheehan DV, Lecrubier Y, Sheehan KH, Amorim P, Janavs J, Weiller E, et al. The MINI Plus-International Neuropsychiatric Interview (M.I.N.I.): the development and validation of a structured diagnostic psychiatric interview for DSM-IV and ICD-10. J Clin Psychiatry. 1998;59 Suppl 20:22-33.

Sumich, A.L., Kumari, V., Heasman, B.C, Gordon, E and Brammer, M.J (2006). Abnormal asymmetry of N200 and P300 event-related potentials in subclinical depression. *Journal of Affective Disorders*, 92: 171 - 183.

The WHOQOL Group (1998). Development of the World Health Organization WHOQOL-BREF Quality of Life Assessment. Psychological Medicine, 28: 551-558 Cambridge University Press doi:10.1017/S0033291798006667

Tunis SR, Stryer DB, Clancy CM (2003). Practical trials: increasing the value of clinical research for decision making in clinical and health policy. JAMA, 290: 1624-1632.

Venn HR, Watson S, Gallagher P, Young AH (2005). Facial expression perception: an objective outcome measure for treatment studies in mood disorders? *International Journal of Neuropsychopharmacology*, 9, 1–17.

Williams LM, Kemp A, Felmingham K, Liddell B, Palmer D & Bryant RA (In Press). Negativity biases to overt and covert signals of fear: Dissociation by trait anxiety and depression. *Journal of Cognitive Neuroscience* (In Press).

54. Williams LM, Simms E, Clark CR, Paul RH, Rowe DL, Gordon E (2005). The testretest reliability of a Standardised neurocognitive and neurophysiological test battery: “NeuroMarker”. *Int J Neurosci* 115:1605–1630.

55. Zhang, X., Gainetdinov, R.R., Beaulieu, J.M., Sotnikova, T.D., Burch, L.H., Williams, R.B., Schwartz, D.A., Krishnan, K.R.R and Caron, M.G (2005). Response to Correspondence: Loss-of-Function Mutation in Tryptophan Hydroxylase-2 Identified in Unipolar Major Depression. *Neuron*, 48: 705-706.

Kadouri, A., Corruble, E., and Falissard, B. (2007) The improved Clinical Global Impression Scale (iCGI):development and validation in depression. BMC Psychiatry, 7:7.

Trivedi, M. H., Rush, A. J., Ibrahim, H. M., Carmody, T. J., Biggs, M. M., Suppes, T., et al. (2004). The Inventory of Depressive Symptomatology, Clinician Rating (IDS-C) and Self-Report (IDS-SR), and the Quick Inventory of Depressive Symptomatology, Clinician Rating (QIDS-C) and Self-Report (QIDS-SR) in public sector patients with mood disorders: a psychometric evaluation. *Psychol Med, 34*(1), 73-82.

12. PROTOCOL APPROVAL

_______________________________ ________

Signature Date:

Dr. Leanne Williams

Director

Brain Dynamic Centre

_______________________________ ________

Signature Date:

Deborah Kargl

Global Trial Manager

BRC Operations Pty. Ltd.

13. INVESTIGATOR SIGNATURE PAGE

BRC Operations PTY LTD.
Level 12, 235 Jones Street
Ultimo, NSW 2007

Tel: +61 (0)2 9211 7120
Fax: +61 (0)2 9211 2710

International Study to Predict Optimised Treatment – in Depression

ISPOT - D

Investigator statement

I have read the protocol, including all appendices, and I agree that it contains all necessary details for me and my staff to conduct this study as described. I will conduct this study as outlined herein and will make a reasonable effort to complete the study within the time designated.

I will provide all study personnel under my supervision copies of the protocol and access to all information provided by BRC Operations Pty. Ltd. I will discuss this material with them to ensure that they are fully informed and trained about the study.

Principal Investigator Name (Printed)	Signature
Date	

14. APPENDICES

Appendix A: The Standardised Integrative Methodology used in iSPOT

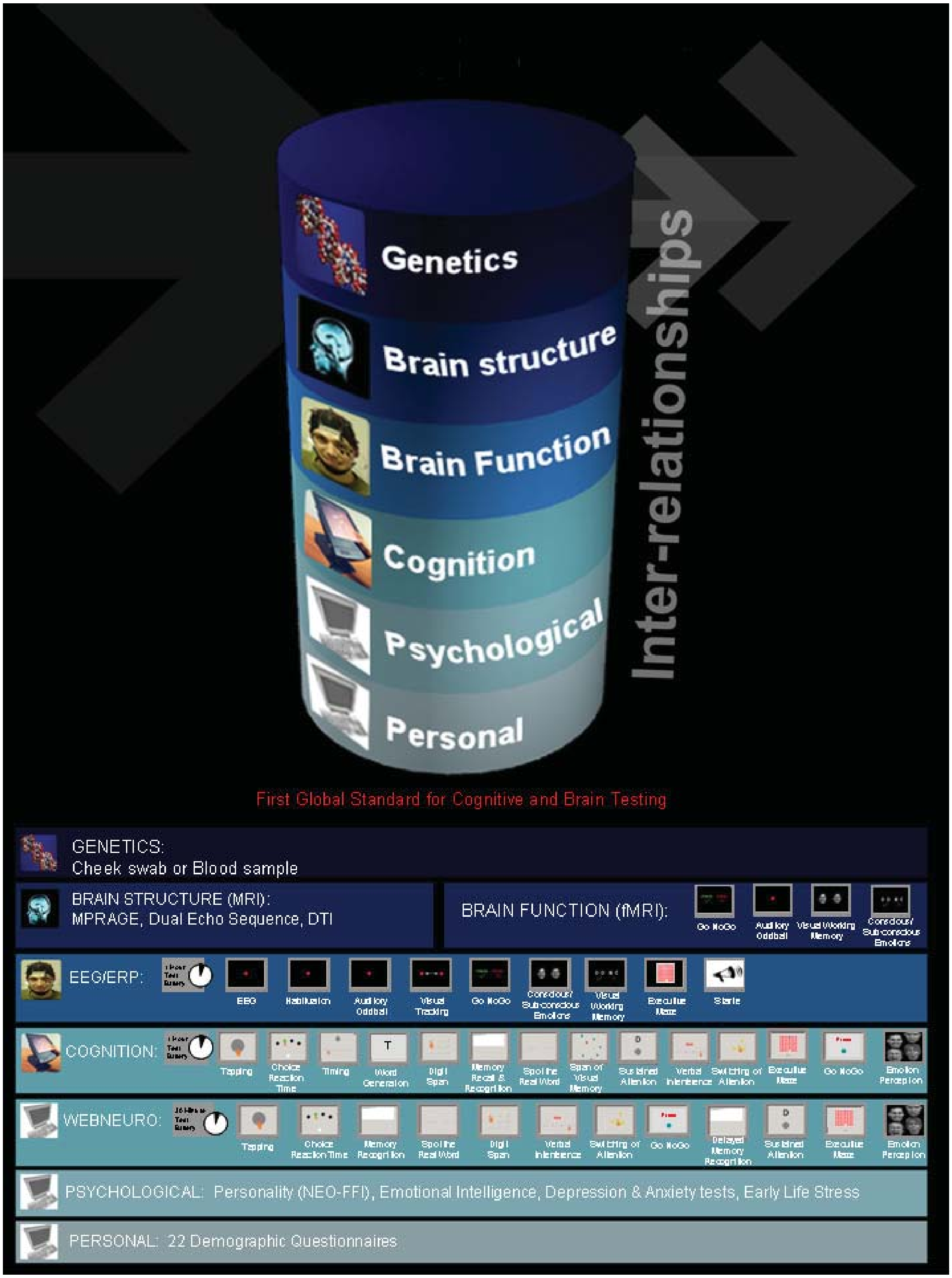

Appendix B: The Neuropsychology (Cognition) Test Battery

Motor Tapping

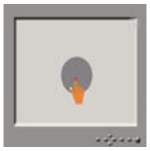

The subject is required to tap a circle on the touch screen, with their index finger, as many times as possible in thirty seconds. This is repeated for both hands.

Functions measured: basic motor function, hand eye coordination, fine movement speed and manual dexterity.

Practical significance: Everyday motor skills such as typing and machine operation.

Choice Reaction Time

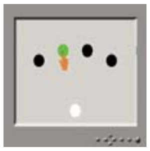

One of four circles lights up, in different positions on the touch-screen. The subject is required to press the lit circle as quickly as possible.

Functions measured: visuomotor coordination, the speed and accuracy of selecting an appropriate response, and the trade-off between speed and accuracy.

Practical significance: visual discriminative judgment and response, basic sensori-motor and decision-making functions. Examples: visual monitoring tasks requiring choice and reaction such as air traffic control, driving judgment.

Time estimation

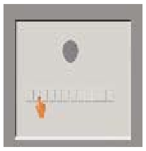

A circle appears on the screen for 1 to 12 seconds, after which time, the subject is required to indicate the correct duration of the circle's appearance by choosing from a number of possible time options.

Functions measured: reflects the ability to accurately estimate time duration. This requires attention and working memory processes.

Practical significance: relates to the ability to preplan actions that constitute purposeful behavior, deciding their temporal onset, and monitoring their time course once they have been initiated. Also relates to general time organization skills involved with effective timing of decisions, and anticipating outcomes.

Span of Visual Memory

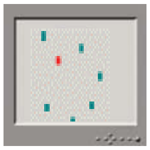

Squares on the touch screen light up in a random order. Four seconds later, the subject hears a tone indicating they have to reproduce, by pressing the squares, the order in which the squares previously lit up.

Functions measured: aspects of working memory including the capacity to hold and sequence visuo-spatial information in short-term memory, and maintain attention.

Practical significance: Ability to hold and retain new spatial information. Skills crucial to most everyday, non verbal tasks requiring memory. Examples include navigation and operating industrial machines.

Digit Span

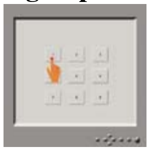

The subject hears a series of digits and is then immediately asked to enter the digits on a numeric keypad on the touch screen, either in forward or reverse order. The number of digits in each sequence will gradually be increased from 3 to 9. The score is the maximum number of digits the subject can reliably repeat without making mistakes.

Functions measured: Short term verbal memory (Score Forwards), working memory operations (Score Backwards).

Practical significance: Ability to add, retain and operate on new verbal information. Skills crucial to most everyday, verbal tasks requiring memory. Everyday examples include remembering telephone numbers and shopping lists.

Memory Recall and Recognition

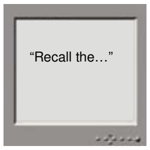

There are two parts to this test. In the recall part, the subject is presented with a list of 12 words, which they are asked to memorise. The list contains 12 concrete words. Words are closely matched on concreteness, number of letters and frequency. The list is presented 4 times in total, and the subject is required to recall as many words as possible after each presentation. Answers are recorded through a microphone into '.wav' files. The subject is then presented with a list of distracter words and asked to recall those. After this, the subject is asked to recall the 12 words from the original list. Twenty-five minutes later, the subject is again asked to recall the 12 words from the original list. This test assesses the verbal memory recall of the subject.

In the recognition part of the test, the subjects' recognition of the previously presented words are tested. The subject is presented with a series of words on the screen (some of which appeared in the original list) and is then asked to respond 'yes' or 'no' as to whether the word was in the original list.

Functions measured: Ability for new auditory verbal learning, memory recall and recognition, and verbal self-monitoring.

Practical significance: Ability to learn and remember new tasks and skills based on verbal information. This is critical to everyday skills in analyzing information, problem identification, and testing assumptions and interrelatedness of information.

Verbal Interference

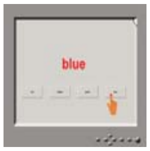

There are two parts to this test. In the first part, the subject is required to indicate the color that the written word spells (and not the incongruent color that the word is written in). In the second part of the test, the subject will be asked to name the color a word is written in (and ignore the actual written word).

Functions measured: The first part measures reading speed and accuracy for individual words. The second part measures the ability to inhibit inappropriate well-learned impulsive automatic responses.

Practical significance: This test assesses the ability of the subject to suppress unwanted 'impulsive', well-learned, automatic responses. This relates to cognitive flexibility and also behavioural control e.g. control of anger.

Spot the Real Word

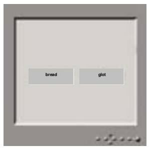

A word and non-sense word pair are presented on the touch-screen. The subject is required to indicate which is the 'real' word by pressing the touch-screen.

Functions measured: assesses language recognition and comprehension and related vocabulary skills.

Practical significance: general language skill, effective writing ability, and provides an estimate of pre-morbid intelligence.

Word Generation

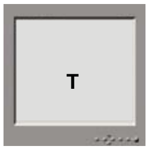

The subject names as many words as possible, in the space of a minute, which begins with a certain letter (F, A and then S in one version of the test). Subjects will be instructed not to use proper nouns, nor to make variations on the same word stem (for example, ‘run’ and ‘running’). The subject will then be asked to name as many animals as possible. The score on the test is simply the number of words generated.

Functions measured: Verbal fluency, an individual’s capacity to produce a sustained stream of spontaneous speech.

Practical significance: Ability to generate and articulate thoughts and ideas in a systematic manner.

Sustained Attention (visual working memory)

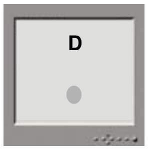

A series of letters are presented on the screen, one by one. The subject is required to press a response button if the same letter appears twice in a row, and at no other time.

Functions measured: The ability to sustain attention over an extended period of time, as well as the ability to update information held in the verbal short-term stores of working memory (in order to detect targets.)

Practical significance: Ability to detect and respond to significant change and ignore irrelevant information under conditions requiring vigilance. Fundamental everyday skills e.g. train, plane, automobile, computer and equivalent machine operations.

Switching of Attention

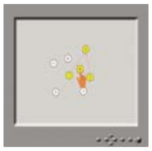

This test contains two simple tests of attention. In the first test, 25 numbers (presented inside circles) are displayed on the screen. The subject is required to press the numbers in ascending sequence (i.e. 1-2-3- etc). This tests the basic ability to hold attention on a simple task. The second test will require the subject to connect numbers and letters in an ascending, but alternating, sequence (i.e. 1-A-2-B-3-C etc). The numbers 1-13 and the letters A-L are presented in circles on the touch screen.

Functions measured: Parts 1 and 2: Visuomotor tracking, simple attention. Part 2 only: Ability to shift the course of ongoing mental activity.

Practical significance: Part 1: Simple ability to attend. Part 2: Ability to sustain and control the direction of attention. Critical activity for everyday multitasking skills e.g. management, driving.

Executive Maze

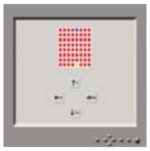

A grid of circles appear on the computer screen. The subject is required to find the hidden path through the grid, from the beginning circle at the bottom of the grid to the end circle at the top. Using a directional button box, the subject navigates across the grid to discover (by trial and error) a hidden pathway linking the circles from the start to the end of the maze. One tone (and a red cross at the bottom of the screen) is presented if the subject makes an incorrect move, and a different tone (and a green tick at the bottom of the screen) if they make a correct move. Each session the subject does the task, the path in the maze is the same (it changes between sessions). Once the subject reaches the end circle within a session, they are required to repeat the (still hidden) maze from start to finish, as many times as possible until the task ends.

Functions measured: Planning, abstraction, foresight, error correction, the ability to choose, try, reject and adapt alternative courses of thought and action; visuospatial learning and memory. Reflect the capacity of executive functioning as well as the flexibility of visuospatial processing and sensori-motor responding.

Practical significance: Assesses the ability to plan strategically to solve a complex practical problem. It involves both planning ahead and monitoring and correcting errors (error monitoring) to meet these plans. Completing the maze relies on the generation of response options, trial-and-error adaptation and the ability to choose an alternative course of action as required by the context. The strategic planning skills, ability to adapt and the flexibility to choose alternative courses of action, assessed by this task are essential to organising ability, creativity and innovation.

Go NoGo

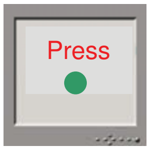

The color of the word 'PRESS' will be frequently presented in green (Go) and infrequently in red (NoGo). The subject will be required to inhibit circle-tapping responses on red. This task measures target detection rate, response time, errors of commission and omission.

Functions measured: inhibition - the capacity for suppressing for suppressing well-learned, automatic responses. The ability to re-initiate response after response inhibition which requires sustained attention and behavioural flexibility.

Practical significance: Effective in assessing impulsivity (elevated commission error rates), or inattention (elevated omission error rates) in those with attentional problems. These relate to risk-taking tendencies, focus on tasks requiring sustained cognitive effort and social relationship skills.

Emotion Recognition and Recall Task

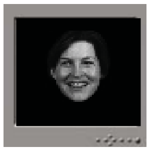

Subjects are presented with faces expressing different basic emotions (happiness, sadness, fear, anger, disgust, neutral), one at a time on the computer screen, and asked to identify the specific emotion associated with each face within a multiple-option response format. The delayed recall component tests memory for prior targets against foils, as a further test of implicit emotional processing and emotional biases.

Functions measured: basic emotion recognition and discrimination between emotions, memory for emotional expression.

Practical significance: Emotion recognition ability is a key aspect of social cognition, relating to effective social interaction and empathy. Better and faster recognition is associated with higher emotional intelligence, specifically empathy.

Appendix C: The Electrical Brain Function Test Battery

Resting EEG

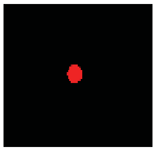

Subjects are asked to rest quietly and focus on the red dot (eyes open) and then repeat with eyes closed (whilst imagining/visualizing the red dot). The baseline EEG measure allow for comparison between resting and active states of the brain.

Auditory Oddball

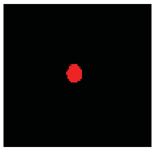

Subjects are presented with a series of high and low tones, at 75dB and lasting for 50ms (with rise and fall times of 5ms). They are instructed to ignore the low ('background') tones (presented at 500Hz) and to press, with the index finger of each hand, a response button only when they hear high infrequent ('target') tones, which will be presented at 1000Hz. Speed and accuracy of response are equally stressed in the task instructions. The task allows for assessment of processing novel task relevant, whilst ignoring task irrelevant, information.

Go-No Go

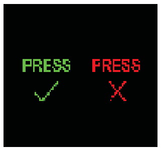

Subjects are repeatedly presented with the word 'PRESS' (for 500ms) on the screen. Subjects are instructed to press a response button, with the index finger of each hand, if the word appears in the color green, but to not respond if the word appears in red. Speed and accuracy of response are equally stressed in the task instructions. This task tests the executive functions of the pre-frontal and orbito-frontal cortex, in particular the ability to inhibit or suppress well-learned and inappropriate automatic responses.

Facial Emotion perception

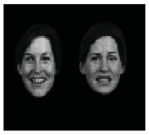

In this task, faces are presented consciously and nonconsciously (subliminally). *Unconscious*: Subjects will be told they will see a series of different faces presented in pairs, but that the first face of each pair will be presented so briefly as to be barely visible. They will be told that they need to pay attention, as they will be asked about the faces later on. *Conscious*: Subjects are told that they will see a different series of faces, but that these are presented only one at a time. Again, they are instructed to pay attention to the faces because they will again be asked about them later on. This task assesses brain and body perception of faces showing emotion (the face stimuli are from the 'Gur' set of emotions).

Visual Working Memory

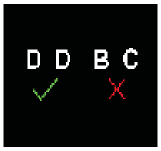

This task consists of a series of letters presented to the subject on the computer screen. If the same letter appears twice in a row (i.e. a 'target letter'), the subject is required to simultaneously press response buttons with the index finger of each hand. Speed and accuracy of response are equally stressed in the task instructions. In addition, intermittent checkerboard stimuli elicit 'novelty P300a' visual ERPs. The task is designed to assess sustained attention and working memory.

Prepulse Inhibition

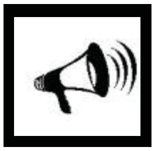

In this task, the subject is presented with a series of acoustic startles (noise burst of 50ms at 100dB, instantaneous rise and fall). This sound is designed to elicit a startle ('fight or flight') response, which is traditionally measured via the eye-blink reflex (although the full profile of brain-body measures will also be examined). Successive stimuli will be separated by a random interval between 10 and 15 seconds. Some startle stimuli will be preceded by 50ms with a pre-pulse, which will consist of quieter noise burst (P: 20ms at 75dB with a 5ms rise and fall time). This pre-pulse has the effect of inhibiting the startle response, and can be used to measure sensory gating mechanisms in the subject. The sequence is fixed and will be presented, without a break, as follows: C, P, C, P, P, C, C, P (Block 1) and P, C, P, C, C, P, P, C (Block 2).

Appendix D: MRI and fMRI

MRI

T1 Mprage sequence

Saggital orientation.

Slice thickness =	1	mm.
No. slices	=	180 (no gap).
Flip angle	=	12 .
TR	=	9.7 ms.
TE	=	4.
TI	=	200.
Matrix	=	256x256.
FOV	=	256 mm x 256 mm.
Pixel sise	=	1.00 x 1.00.
NEX	=	1.

A DTI acquisition will also be undertaken.

Functional MRI

Saggital orientation.

Slice thickness =	3mm.	
No. slices	=	43 (no gap).
Flip angle	=	90 .
TR	=	2.5sec.
TE	=	40ms.
Matrix	=	64 x 64.
FOV	=	24 mm x 24 mm.

Functional MRI tasks:
Four tasks equated to those used with ERP recording are undertaken:

- Go-NoGo (inhibition).
- Auditory Oddball.
- Working Memory (n-back).
- Facial Emotion Processing.

Appendix E: Markers for first pass analysis

The list of markers used in the initial analyses are as follows (further post hoc exploratory analysis will include the most significant additional markers):

Screening

Life history information is assessed only once, and is not susceptible to change with medication. Thus, these measures will be used as covariates where appropriate, or to group subjects.

Covariates include:
Exposure to traumatic and/or stressful events.
Medical history.
History of birth complications.

General Cognition (34 Measures)

The cognitive markers are listed for each test as follows:

Memory Recall and Recognition: Delayed Recall, Immediate Recall, Intrusion Errors, Recognition Memory.

Auditory Oddball: Reaction Time, False Positive Errors, False Negative Errors, Response Variability.

Go No-Go: Reaction Time, False Positive Errors, False Negative Errors, Response Variability.

Executive Maze: Total Overrun Errors, Completion Time.

Sustained Attention (Visual Working Memory): Reaction Time, False Positive Errors, False Negative Errors, Response Variability.

Sensorimotor Tapping: Dominant hand number of taps, Non-Dominant hand number of taps.

Choice Reaction Time: Average Reaction Time.

Span of Visual Memory: Span Total Score.

Digit Span: Forward Span Score, Backwards Span Score.

Visual/Verbal Interference: Visual Interference Accuracy Score, Visual Interference RT, Verbal Interference Accuracy Score, Verbal Interference RT.

Switching of Attention: Part I Completion Time, Part I Errors, Part II Completion Time, Part II Errors.

Word Generation: Word Fluency, Animal Category Fluency.

Social Cognition (13 Measures)

The battery of tests tapping aspects of social cognition produces 13 test marker scores which are listed for each test as follows:

NEO-FFI: Five dimensions of personality traits: Openness, Conscientiousness, Extraversions, Agreeableness and Neuroticism.

Emotional Intelligence (Brain Resource Inventory of Emotional intelligence Factors, BRIEF; Kemp et al., 2005b), with the key factor scores for Social/Relationships, Empathy/Intuition and Self-Esteem.

Depression Anxiety and Stress Scale: Depression, Anxiety, Stress.

Emotion Recognition Test: Accuracy and Reaction time for recognizing basic facial expressions of emotion.

ERPs: **(90 Measures)**

Event-related potentials (ERPs) index brain activity elicited by activation tasks. Data reduction will occur in the following steps:

Artefact correction of raw electrical brain data using Brain Resource's automated correction algorithms.
Scoring of peak ERP components for each activation task using Brain Resource's semi-automated scoring algorithms.
Confirmation of scoring validity via visual inspection.

The core ERP markers for five tasks of interest are as follows:

Working Memory: P150 and P450 amplitude and latency for target (sustained attention) and background (working memory) stimuli [2 components x 2 properties x 2 conditions = 8 measures].

Auditory Oddball: N100, P200, N200 and P300 amplitude and latency for target (task-relevant selective attention) stimuli and N100, P200 amplitudes and latency for background (task irrelevant) stimuli [4 components x 2 properties targets plus 2 components x 2 properties backgrounds = 12 measures].

Go No-Go: N100, P200, N200 and P300 amplitude and latency for No-Go (inhibition) stimuli [2 components x 2 properties x 2 conditions = 8 measures].

Facial Emotion: P80, N120, VPP, N200 and P300 amplitude and latency for each facial emotion (Happy, Sad, Fear, Anger, Disgust and Neutral) [5 components x 2 properties x 6 emotions = 60 measures].

Novelty: P300a amplitude and latency for distracter (novelty) stimuli [1 component x 2 properties = 2 measures].

EEG: **(12 Measures)**

Resting: Power for Alpha, Beta, Theta, Delta.

EEG Asymmetry: for each of the above bands.

EEG synchrony: for each of the above bands.

Autonomic: **(16 Measures)**

Heart Rate: Average BPM and Variability during Working Memory, Oddball, Go NoGo, Facial Emotion tasks, and Resting (Eyes Open, Eyes Closed).

Skin Conductance: Number of Skin Conductance responses elicited during the Working Memory, Oddball, Go NoGo, Facial Emotion tasks.

Genetics
Genetic data analysis involves the extraction of genotype information from DNA samples. We will focus on candidate polymorphisms for Depression, which include those with some existing support from published studies:

BDNF Val66Met (Met allele),
SERTPR (5HTT short allele),
HTR1-*C*1019 G (5HT1A),
T102C (5HT2A),

BCL-2,
TPH2 (G1463A),
MTHFR C677T.

Array analyses will be used to extract multiple additional genotypes (and further analysis will be undertaken of gene expression, proteomics and other molecular analyses) not yet associated with these disease groups in the published literature, for exploratory analyses.

MRI (structural and functional)

Structural and functional MRI data will be acquired and analysed for ten percent of the subjects in this study. It will thus be analysed separately (following data reduction) for these subjects, and then in combination with the other markers, using the same planned analyses as outlined above, That is, for the subset of subjects with all data, MRI data will be included in analyses with all other measures to determines its contribution to profile Disease and Treatment Markers.

Data reduction for structural MRI Analyses: Voxel-based morphometry with the statistical software SPM2 (http://www.fil.ion.ucl.ac.uk/spm/software) will be used to quantify gray matter volume for regions of interest (Ashburner and Friston, 2000; Good et al., 2001). ANOVA (within a regression model) can then be undertaken to determine whether groups differ on grey matter, using the same statistical model as for other markers. We will generate structural MRI markers by quantifying grey matter volume for all regions of interest using a standardised set of masks based on neuroanatomical divisions and defined by the Automated Anatomical Labeling (AAL) protocol (Tzourio-Mazoyer et al., 2002).). One SPM2 has been used to generate these volumes, they are expressed in numerical form and may thus be analysed using the same procedures as for other markers. Markers will include grey matter for the following regions:
Medial prefrontal cortex (encompassing the anterior cingulate cortex, BA24/32, and medial orbital to superior frontal structures, extending to BA9/10, separated for dorsal and ventral portions).
Lateral prefrontal cortex (both dorsal and ventral divisions),
Parietal cortex (both inferior and superior divisions),
Occipital cortex (focusing on inferior and medial occipital portions),
Temporal cortex (superior, inferior and middle temporal portions bounded by these gyri), *Thalamus,*
Amygdala,
Hippocampus,
Basal ganglia (encompassing caudate, putamen, and nucleus accumbens),
Brainstem (defined by upper midbrain).

DTI analysis will be undertaken based on predicted network dysfunctions.

Data reduction for functional MRI Analyses:
Preprocessing and statistical analysis will be implemented within the statistical parametric mapping software package SPM2. A priori search regions of interest (ROIs) will be defined using the same protocol as for structural MRI. Activated voxels within each ROI will be identified for the contrast of relevance to each activation task, usinge an alpha level of $p < 0.05$ (with small volume correction) and a spatial extent of at least 20 voxels per cluster. For regions showing significant activation, the most activated voxels will be identified and the time series of Blood-oxygen-level-dependent (BOLD) signal extracted. This signal is expressed as a time series from which a difference score (activation – baseline) can be computed. This computation yields a single value (percent signal change) which can be used in planned analyses in the same way as other markers.

Prior analyses and biophysical modelling with the Brain Resource International Database have established an integrative link between functional MRI and ERPs derived from the same activation tasks (Robinson et al., 2006), providing a platform from which to interpret the profile of Markers identified when these sources of data are combined in the planned analyses.

fMRI percent signal change markers will be generated for each of the above regions for the following activation tasks, corresponding to those for ERPs.

Working Memory: Percent signal change for each region for target (sustained attention) versus background (working memory) stimuli.

Auditory Oddball: Percent signal change for each region for target (task-relevant selective attention) stimuli versus background (task irrelevant) stimuli.

Go No-Go: Percent signal change for each region for Go (response speed) versus No-Go (inhibition) stimuli.

Facial Emotion: Percent signal change for each region for each facial emotion (Happy, Sad, Fear, Anger, Disgust) versus Neutral.

Novelty: Percent signal change for each region for distracter (novelty) versus baseline stimuli.
