# Supplementary material for: A Cognitive Biotype of Depression and Symptoms, Behavior Measures, Neural Circuits, and Differential Treatment Outcomes: A Prespecified Secondary Analysis of a Randomized Clinical Trial
Source: JAMA Netw Open. 2023 Jun 15;6(6):e2318411. doi: 10.1001/jamanetworkopen.2023.18411 (PMC10273022; doi:10.1001/jamanetworkopen.2023.18411)
Supplement: Supplement 2. — eMethods. Supplemental Methods eResults. Supplemental Results eTable 1. Demographic and Clinical Characteristics of Participants at Baseline eTable 2. Neurocognitive Domains, Tests, and Descriptions eFigure 1. CONSORT Diagram eFigure 2. Scree Plot of the k-means Clustering Solutions with Different Numbers of Clusters eFigure 3. Posttreatment Cognitive Performance for Composite Measures in Cognitive Biotypes eReferences. [file jamanetwopen-e2318411-s002.pdf]

Supplemental Online Content

Hack LM, Tozzi L, Zenteno S, et al. A cognitive biotype of depression and symptoms, behavior measures, neural circuits, and differential treatment outcomes: a prespecified secondary analysis of a randomized clinical trial. *JAMA Netw Open*. 2023;6(6):e2318411. doi:10.1001/jamanetworkopen.2023.18411

eMethods. Supplemental Methods

eResults. Supplemental Results

eTable 1. Demographic and Clinical Characteristics of Participants at Baseline

eTable 2. Neurocognitive Domains, Tests, and Descriptions

eFigure 1. CONSORT Diagram

eFigure 2. Scree Plot of the k-means Clustering Solutions with Different Numbers of Clusters

eFigure 3. Post-treatment Cognitive Performance for Composite Measures in Cognitive Biotypes

eReferences

This supplementary material has been provided by the authors to give readers additional information about their work.

© 2023 Hack LM et al. *JAMA Network Open.*

eMethods. Supplemental Methods

Overview and Patients

Race and ethnicity categories were provided by the investigator and participants classified themselves. Race and ethnicity were assessed in this study to determine if outcomes varied by these measures.

Clinical inclusion criteria were diagnosis of MDD according to DSM-IV criteria using the structured clinical Mini-International Neuropsychiatric Interview[1], a score of >=16 on the 17-item Hamilton Rating Scale for Depression ($HRSD_{17}$)[2] and performance within prior established healthy limits on the Spot-the-Word Test[3] estimate of premorbid intellectual functioning[4]. Exclusion criteria were bipolar disorder, any psychosis, OCD, PTSD, eating disorder, substance dependence, head injury, sensory impairment and/or medical (including neurological) disorder contraindicated by the protocol. Patients were free from pharmacotherapy, behavioral therapy, and other forms of therapy (such as St John's Wort) at baseline[5].

Psychometric Validation of the Neurocognitive Test Battery

The IntegNeuro tests used in this study have undergone extensive construct validation using principal component analyses (PCA) in two large healthy norm samples and in one large clinical sample. These analyses demonstrate that the composite scores listed in **eTable 2** are largely independent from each other. The results of the PCAs described below lead us to include nine cognitive domains for the current analyses:

First Healthy Sample

1316 healthy participants who were recruited for the norming of Computerized Integneuro cognitive tests, and 1229 completed cognitive testing (50.5% females; age range: 16 to 60 years; mean age = 32.77, SD = 12.95)[6]. PCA was first undertaken with a subgroup of 410 subjects, who had complete datasets of composite scores on all general cognitive tests, except GoNoGo, which was not administered. We used an obliminal rotation (direct obliminal, with delta = 0). A larger set of subjects (n = 891, who had 5% missing data on the verbal memory test) were used as a second subgroup to perform a confirmatory PCA. In this confirmatory PCA, we focused on confirmation of factor structure for those factors which were not defined by loadings from the verbal memory. The PCA for both groups revealed the same underlying structure. PCA for the subgroup of 410 subjects with complete data revealed eight component factors with rounded eigenvalues of 1.0 or greater, and which together accounted for 80.36% of the variance in test scores. The first factor was a general cognitive "g" factor which was unrotated and accounted for 20.35% of variances across tests reflecting overall cognitive performance.

The other seven factors were defined by the following composites (and test scores):

1. Information Processing Speed (Switching of attention Parts 1 and 2 completion time, Verbal interference name word and name color accuracy, Choice reaction time)
2. Verbal Memory (Verbal Memory test scores for immediate recall, delay recall and recognition)
3. Working Memory Capacity (Total and maximum span for forwards and reverse Digit Span recall)
4. Sustained Attention (Accuracy and Reaction time for the Continuous Performance N-back test)
5. Psychomotor (or sensorimotor) Function (number of taps and pause between taps)
6. Verbal Processing (Verbal fluency for letters F, A, S and animal category fluency)
7. Executive Function (Completion time, Errors and overrun errors on the Maze test)

Second Healthy Sample

In a subsequent independent norm sample of 1,000 healthy subjects (465 males, 535 females), aged 6–91 years (mean = 41.08, SD =23.04)[7], the GoNoGo test was included in analyses. PCA confirmed the original norm sample structure and identified a specific additional factor:

1. Response Inhibition/Impulsivity (Reaction time and accuracy on the GoNoGo test).

In this sample the factor components accounted for 73.38% of variance in the test scores and a general 'g' factor (beyond the combined loading of tests on information processing speed) was not observed. Further, this factor structure in the second norm sample was verified in the subset of adults aged 18-59 years.

© 2023 Hack LM et al. *JAMA Network Open.*

Clinical sample
A clinical sample of 160 participants with a primary diagnosis of depression and associated diagnoses of anxiety disorders were assessed on the same cognitive measures[8]. In this sample PCA with oblimin rotation revealed factors equivalent to those in the second normative sample, accounting for 84.2% of the variance. We note that an additional factor was identified due to verbal interference test scores loading together and splitting off from information processing speed, as follows:

1. Cognitive flexibility (or interference) (name color and interference scores on the Verbal Interference test).

The above factor components were used as the foundation for subsequent generation of composite scores (which are an average of the individual test scores that load on each component/domain (eTable 2) used in the present study. We note two variations included in the present study. First, we did not have data available for iSPOT-D subjects for the Verbal Fluency tests since these tests rely on manual scoring of 'wav' file recordings. Thus, we did not include a 'verbal processing' composite equivalent to the verbal processing component identified in the original norm sample. Second, we entered the Choice Reaction test as its own "Decision Speed" composite distinct from the Information Processing Speed composite, because decision speed is not as well distinguished by RDoC constructs as is information processing speed.

Functional Capacity Assessment

In addition to the Social and Occupational Functioning Assessment Scale (SOFAS)[9], we also assessed functional capacity using the World Health Organization Quality of Life (WHOQoL) domains of physical health, psychological health, social relationships, and environment rated 1-4[10].

Neuroimaging

Structural Scan
A high-resolution T1-weighted structural scan was acquired for registration of functional images. A total of 180 contiguous slices, each 1 mm thick, covered the whole brain with an in-plane resolution of 1 mm x 1 mm. It was acquired in the sagittal plane using a 3D spoiled gradient echo (SPGR) sequence (TR = 8.3 ms; TE = 3.2 ms; flip angle = 11 degrees; TI = 500 ms, NEX = 1 and ASSSET = 1.5; frequency direction: S/I; matrix = 256 x 256, 180 contiguous slices, 1mm isotropic voxels).

Go-NoGo Task
Cognitive control was assessed using a Go-NoGo task. 'Go' trials (the word "press" in GREEN), required subjects to respond as quickly as possible, while in the 'NoGo' trials ("press" in RED) subjects were to withhold responses. 180 Go and 60 NoGo stimuli were presented in pseudorandom order; 500 ms each with an interstimulus interval of 750 ms.

Imaging Acquisition
Head motion was restricted with foam pads and subject alertness was monitored with an eye-tracking system.

Image Pre-processing
Pre-processing and data analysis was performed using Statistical Parametric Mapping (SPM) software implemented in MATLAB (SPM8; Wellcome Department of Cognitive Neurology) and FSL[11] using established protocols[12,13]. Sections of the processing pipeline are within a Singularity environment to ensure reproducibility across computational systems. Briefly, T1-weighted data were normalized to standard space using the FMRIB nonlinear registration tool. For functional images, following removal of the first 3 volumes, images were then motion corrected by realigning and unwarping to the first image of each task run. Quality control involved excluding subjects based on the percentage of time points censored for either frame-wise displacement or variance spikes (maximum 25% time points removed). We also ensured that scans with incidental findings, major scanner artifacts, and signal dropout were not included. Following estimation and then removal of physiological noise from these motion-corrected functional images they were normalized to the Montreal Neurological Institute (MNI) template[14], co-registered to T1 data using FMRIB, smoothed using an 8 mm Gaussian kernel and high-pass filtered using a cutoff period of 128 seconds.

© 2023 Hack LM et al. *JAMA Network Open.*

Quantification of Region of Interest Activation

The procedure we followed to quantify the dysfunction of each region of interest is described in detail here[8]. In summary, the brain regions of interest comprising the cognitive control circuit were defined from the meta-analytic database Neurosynth[15]. These regions were then refined by removing the regions that did not pass quality control or for which circuit quantification did not meet a set of psychometric criteria, such as construct validity, internal consistency, and independence. Of the remaining regions, we only retained those that are also implicated in our theoretical synthesis of dysfunctions in depression and anxiety for a final set of 3 regions of interest for the cognitive control circuit[8,16]. Task-evoked activation was quantified using a generalized linear model analysis in which task events were convolved with a canonical hemodynamic response function as implemented in SPM8. The contrast of interest for the cognitive control circuit was NoGo > Go trials. Activations in the clinical sample were then expressed in standard deviation units relative to mean and standard deviation of a healthy sample acquired on the same scanner (z-scores). This approach to quantifying clinical subject level data drew on our prior work which established the construct validation of such circuit quantification[8].

Statistical Analysis

K-means Clustering

We elected to use the scree plot elbow method and silhouette score with Euclidean distances as metrics for determining the ideal number of clusters because these are commonly used methods. The scree plot for a k-means clustering analysis shows the average within-cluster sum of squares (WSS) or the average sum of squared distances between each point and its assigned centroid for each cluster for different values of k. The optimal number of clusters is often chosen at the "elbow" or bend in the scree plot, where the decrease in WSS slows down and the curve becomes less steep. Silhouette scores represent the mean silhouette coefficient over all instances of the dataset, and they range from -1 to 1. Scores closer to 1 indicate a model with more coherent clusters.

Mediation Models

We used the Preacher-Hayes bootstrapping method for estimating a simple mediation model with a binary predictor X, a continuous mediator M, and an outcome variable Y. This method is based on structural equation modeling (SEM) routines and addresses some limitations of mediation model estimates based on regular regression output. The PROCESS macro was used to implement the Preacher-Hayes method in SPSS[17,18].

A simple mediation model takes the form illustrated below:

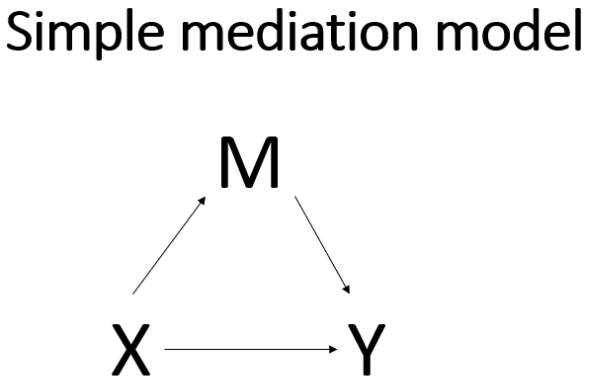

This model is captured in two equations:

© 2023 Hack LM et al. *JAMA Network Open.*

$$M_i = i_M + aX_i + e_{M_i}$$

$$Y_i = i_Y + c'X_i + bM_i + e_{Y_i} \ ,$$

where i denotes case or observation, iM and iY are regression constants, and eMi and eYi are errors in estimation of Mi and Yi.

The path from X to M is called a (equation 1), the path from M to Y is called b (equation 2) and the path from X to Y is called c’ (equation 2). The indirect effect of X on Y through M is obtained by **multiplying a and b (ab). This ab multiplication captures the part of the effect of X on Y that operates indirectly through M**. The remaining component of X’s effect on Y, represented by c′, is the direct effect of X. This direct effect is of less interest in mediation analysis because it is the part of the relationship between X and Y not attributable to the mechanism through M (i.e., everything about the relationship between X and Y except the mediation process that is a part of the model). The total effect is the sum of the direct effect and the indirect effect.

In this simple mediation model, we included the covariates age, baseline $HRSD_{17}$, and family history of MDD. The interpretation of the paths as summarized above remains the same, and the effect is to hold the covariates constant for the interpretation of path coefficients.

The SEM routines implemented in the PROCESS macro address some of the assumptions and limitations of mediation model estimates based on regression models. Regression models such as implemented in the Baron and Kenny approach and/or with the standard Sobel test assume that the sampling distribution of the indirect effect **ab** is normal, an assumption that is met only with large samples. The PROCESS solution, implementing the Preacher-Hayes method, is used to calculate the confidence interval via bootstrapping. The Preacher–Hayes method provides point estimates and confidence intervals by which one can assess the significance or non-significance of a mediation effect. Point estimates reveal the mean over the number of bootstrapped samples and, if zero does not fall between the resulting confidence intervals of the bootstrapping method, one can confidently conclude that there is a significant mediation effect to report.

The bootstrapping method also provides the advantage compared to regression approaches of increasing power.

Mechanism and causality:
The Preacher-Hayes mediation model is designed to address questions about mechanism, extending beyond the simple question of whether or not an effect exists. The mediator, M, can be considered an intervening variable or a mechanism, explaining how a given effect (Y) occurs. Because our design did not allow for a strict temporal precedence between cause and effect our conclusions focus on the consistency of the data with a hypothesis of mediation. We sought to eliminate spurious covariation with variables such as overall symptom severity. We acknowledge some overlap in measures used to allocate X and assess change in M, but not to the extent of a confounder. Future studies are warranted to test stronger causal inferences in a design optimized by experimental manipulation of the cognitive biotype (X) and through establishing an explicit temporal precedence between M and Y. Our putative mediator, cognitive change, is suitable to such designs as it is amenable to both measurement and manipulation.

eResults. Supplemental Results

© 2023 Hack LM et al. *JAMA Network Open.*

Deriving the Cognitive Biotype

The scree plot (**eFigure 2**) indicates an elbow at 2 clusters, after which the line begins to flatten, indicating that additional clusters do not contribute to meaningfully separating the data and suggesting k=2 as the best solution. Silhouette scores represent the mean silhouette coefficient over all instances of the dataset, and they range from -1 to 1. Scores closer to 1 indicate a model with more coherent clusters. k=2 had the highest silhouette score of 0.334, indicating that it is the model with the most coherent clusters. Although the 3-cluster solution had a Silhouette score nearly similar to that of the 2-cluster solution (0.321), it was not selected because it did not add explanatory value (i.e., it split the impaired cluster only by the extent of impairment on the cognitive measure of executive function and this split was not statistically significant). Silhouette scores for k=4-10 clusters were all lower than k=2, ranging from 0.141 to 0.21.

To verify that k=2 was optimal as compared to k=1, we adapted the procedure proposed by Dinga and colleagues[19] to test how likely an observed average silhouette score is to occur under the null hypothesis of no clusters, i.e. of the data coming from a multinormal distribution. We conducted 10,000 simulation runs, in which we drew 1,008 participants from a multinormal distribution having the same mean for each domain as our data as well as their same covariance. These simulated participants were then used as input in the same k-means clustering procedure as described in the Methods, setting k=2 and the average silhouette score of the solution was calculated. Thus, we obtained a null distribution for this average silhouette score, comprising of 10,000 observations. Finally, we calculated the proportion of average silhouette score generated under the null that were greater than the one we obtained from our data (p-value). This simulation showed that our solution significantly outperformed the null-hypothesis of no clusters in the data (mean null silhouette = $-2.13082*10^{-5}$, p~0).

We also further validated our clustering solution by quantifying its consensus to one obtained using a hierarchical clustering algorithm using Euclidian distance and the Ward method. As a measure of consensus between the cluster assignments between the two algorithms, we use the adjusted Rand index (ARI), the corrected-for-chance version of the Rand index (ARI=0 corresponds to chance, ARI=1 is a perfect match, ARI<0 is a result worse than chance). For our 2-cluster solution, we had an ARI of 0.51. In particular, 66% of participants assigned to the cognitive biotype positive subgroup by k-means were assigned to the same cluster by hierarchical clustering. Similarly, 99% of participant assigned to the cognitive biotype negative subgroup by k-means, were assigned to the same cluster by hierarchical clustering. The balanced accuracy for the hierarchical clustering to reproduce the same results as the k-means clustering was 82%.

Cognitive Biotype and Baseline Functional Capacity

The cognitive biotype was also distinguished by worse WHOQoL physical quality of life (*P*=.003; *d*=-0.22 [95% CI, -0.37 to -0.08]) but slightly better psychological quality of life (*P*=.04; *d*=0.15 [95% CI, 0.004 to 0.30]).

Other Considerations

Although BMI was higher in the cognitive biotype (mean=29.45) than intact cognition subgroup (mean=27.14; *P*<.0001; *d*=0.33), but this difference was small and both subgroups were within the overweight range (25 to 29.9) on average.

Anxiety was also higher in the cognitive biotype (mean=9.53) than the intact subgroup (mean=8.51; *P*=.04; *d*=0.15) but this difference was one symptom point and both subgroups were within the mild range on average. Clusters also did not differ in prevalence of generalized anxiety, social anxiety, panic, specific phobia, or agoraphobia disorders (*P*'s>.05).

Cluster subgroups did not differ on brain morphometry volume metrics for any of the 120 regions assessed, including dLPFC and dACC (*P*'s>.05).

Cognitive Biotype and Baseline Neural Circuit Function

The global cognitive control circuit clinical score, which includes both activation and connectivity, was also reduced in the cognitive biotype positive subgroup (*P*=.038; *d*=0.55 [95% CI, 0.31 to 1.07]).

Cognitive Biotype and Treatment Outcomes at 8 Weeks

Functional Impairment Post-treatment. There was an interaction for the total WHOQoL score and pre- versus post-treatment change in psychosocial function such that there was less improvement with treatment in the cognitive biotype positive subgroup than the negative subgroup (P=.013; η_p^2=0.009).

Regression Analyses

The results of the regression analysis show that being in the cognitive biotype positive subgroup was a significant predictor of change in response inhibition post-treatment (β=0.56, 95% CI=0.35 to 0.77, P<.001). Next, while controlling for change in response inhibition, the results of the second regression analysis show that membership in the cognitive biotype positive subgroup was also a significant predictor of change in $HRSD_{17}$ symptoms; however, the confidence interval included a zero crossing (dependent variable (β=-0.45, 95% CI, -2.05 to 0.19, P<.0001)).

For executive function, regression analysis showed that the pre-treatment cognitively impaired group was a significant predictor of change in executive function post-treatment (β=0.86, 95% CI, 0.68 to 1.04, P< .0001). While controlling for change in executive function, the pre-treatment cognitively impaired group was also a significant predictor of change in SOFAS; however, the confidence interval included a zero crossing (dependent variable (β =0.29, 95% CI, -1.86 to 2.46, P<.0001)).

For response inhibition, regression analysis showed that membership in the cognitive biotype positive subgroup was a significant predictor of change in executive function post-treatment (β=0.55, 95% CI, 0.34 to 0.76, P<.0001). While controlling for change in executive function, the pre-treatment cognitively impaired group was also a significant predictor of change in SOFAS; however, the confidence interval included a zero crossing (dependent variable (β=0.78, 95% CI, -1.28 to 2.83, P<.0001)).

Mediation Models

Lack of change in response inhibition was also a significant mediator of the relationship between cognitive biotype status and lack of SOFAS-derived functional improvement (indirect effect a*b=0.70, bootstrapped 95% CI, 0.28 to 1.23) (**Figure 5C**), but the reverse was not true. Although this mediator also had a large effect size of 1.48 in the total effect, this model was not significant (t=1.42, P=.16). Further, lack of change in executive function significantly mediated the relationship between cognitive biotype status and lack of functional improvement (indirect effect a*b=1.18, bootstrapped 95% CI, 0.55 to 1.95) (**Figure 5B**). This mediator also had a large effect size of 1.48 for its contribution to the total effect model but not significantly so (t=1.42, P=.15).

We saw no significant mediation when testing the alternative model. There was no statistically significant direct effect between cognitive biotype and SOFAS improvement (t=0.27, P=.79). Likewise, there was no statistically significant direct effect between cognitive group status and SOFAS improvement (t=0.73, P=.46).

Discussion

Potential treatment options for the cognitive biotype span repurposed existing drugs and non-drug modalities. Examples include (1) the FDA-approved selective alpha 2A receptor agonist guanfacine, known to selectively target the dLPFC[20]; (2) repetitive transcranial magnetic stimulation (rTMS), which is not mechanistically specific but standard FDA-cleared protocols for depression stimulate dLPFC; and (3) cognitive behavior training, which theoretically engages the cognitive control circuit through training that promotes executive function[16,21]. Furthermore, given that deficits in cognitive control are present in other forms of psychopathology, our analysis approach may be applied to other mental health disorders to identify similar cognitive biotypes in these disorders.

© 2023 Hack LM et al. *JAMA Network Open.*

eTable 1. Demographic and Clinical Characteristics of Participants at Baseline

Measure	Total (n = 1008)	Imaging Subsample (n = 96)
Sex, No. (%)		
Female	571 (56.6)	45 (46.7)
Male	437 (43.4)	51 (53.1)
Age, mean (SD), y	37.8 (12.6)	34.5 (13.5)
Education level, mean (SD), y	14.5 (2.8)	14.1 (3.0)
BMI, mean (SD)	27.8 (7.3)	26.3 (5.7)
Race, No. (%)		
Black	167 (16.6)	1 (1.04)
Hispanic Ethnicity	83 (8.2)	1 (1.04)
White	625 (62)	60 (62.5)
Other[a]	212 (21)	34 (35.4)
Unknown	4 (0.4)	1 (1.04)
Symptom severity, mean (SD)		
$HRSD_{17}$ score/52	21.9 (4.1)	21.1 (4.0)
$QIDS\text{-}SR_{16}$ score/27	14.5 (3.8)	17.6 (4.6)
Functional capacity, mean (SD)		
SOFAS/100	55.9 (9.1)	61.0 (8.9)
WHOQoL-Physical/100	51.8 (14.4)	52.7 (13.1)
WHOQoL-Psychological/100	34.6 (13.8)	33.5 (11.9)
WHOQoL-Social/100	38.6 (19.9)	39.3 (20.5)
WHOQoL-Environmental/100	51.7 (15.8)	58.5 (14.5)

Abbreviations: BMI, Body Mass Index; $HRSD_{17}$, 17-item Hamilton Rating Scale for Depression; $QIDS\text{-}SR_{16}$, 16-item Quick Inventory of Depressive Symptomatology – Self-Rated; DASS, Depression Anxiety Stress Scale; SOFAS, Social and Occupational Functioning Assessment Scale; WHOQoL, World Health Organization Quality of Life scale. [a]The "Other" category included pacific islanders, indigenous Australians, and individuals of the Asia and Indian subcontinent. Participants identified themselves into the racial and ethnic categories.

© 2023 Hack LM et al. *JAMA Network Open.*

eTable 2. Neurocognitive Domains, Tests, and Descriptions

RDoC Cognitive System Construct	Paper-and-pencil Neurocognitive Test	Computerized Integneuro Test	Description of IntegNeuro Test	Measures Averaged for Composite Score	Name of Composite Score	Test-Retest Reliability
**Construct**	**Cognitive Control**					
**Subconstruct:** Goal Selection, updating, representation and maintenance	Austin Maze	Maze	Discover by trial and error a maze path; reflecting planning, monitoring feedback, and error correction, thus assessing executive function abilities.	Accuracy (total, overrun errors), completion time	Executive Function	0.73
**Subconstruct:** Response selection, Inhibition	Go-NoGo	Go-NoGo	Press response pad as quickly as possible to ''Go'' (green) trials, and withhold in ''NoGo'' (red) trials. Assessing inhibition versus impulsivity.	Accuracy (total, false positive, false negative errors), Reaction time	Response Inhibition	0.79
**Subconstruct:** Response selection, Inhibition	Stroop	Verbal Interference	Respond as quickly as possible to naming the of color word (ignore color) and then color of each word (ignore name). Assessing suppression of automatic responses.	Accuracy for name word and name color, Interference time for name word minus color	Cognitive Flexibility	0.77
**Construct**	**Declarative Memory**					
**Subprocess:** Learning, delayed recall	California Verbal Learning Test-Revised (CVLT-R), and RCVLT	Verbal Learning and Memory	Learn and then recall lists of 12 words under immediate recall and delayed recall conditions, assessing verbal learning and memory.	Immediate recall (trials 1-4), delayed recall, recognition accuracy	Verbal Memory	0.62
**Construct**	**Attention**					
**Subprocess:** Sustained Attention	Adaptive Rate Continuous Performance Test, Test of Variables of Attention (TOVA)	Continuous Performance Test (CPT)	Sustain attention to a series of letters (D,C,G, or T). Identify when same letter is repeated ('1-back'), assessing ability to sustain attention and to maintain information held in short-term working memory.	Accuracy (total, false positive, false negative errors), Reaction Time	Sustained Attention	0.75
**Subprocess**: Motor action	Corsi Blocks Test	Choice Reaction Time	Respond to one of four circles as they light up, which assesses simple decision-related reaction time.	Average Reaction Time, Variability of Reaction Time	Decision Speed	0.66
	Finger Tapping	Motor Tapping	Tapping index finger as fast as possible for 60 s; assessing sensorimotor response speed.	Number of Taps, Variability of pauses between taps	Psychomotor Function	0.89
**Construct**	**Working Memory**					
**Subconstruct:** Active maintenance, Flexible updating	Weschler Trail Making Test A, B	Switching of Attention Parts 1, 2	Connect a sequence of numbers (Part 1) and then a sequence of alternating numbers and letters (Part 2).	Completion time for Part 1 and Part 2	Information Processing Speed	0.85
	Digit Span —WAIS III	Digit Span	Repeat a series of digits in forward and backward order; assessing working memory.	Total and Maximum recall span for forward and reverse digits	Working Memory	0.78

Test-retest reliability of each computerized Integneuro Test was assessed in a community population of n = 1,980 healthy subjects with age range 6-81 years (mean=40.32, SD=13.63, 67% female). The time between first assessment and follow-up was 8 weeks on average. The composites scores were used as independent inputs into the k-means clustering analysis.

© 2023 Hack LM et al. *JAMA Network Open.*

eFigure 1. CONSORT Diagram

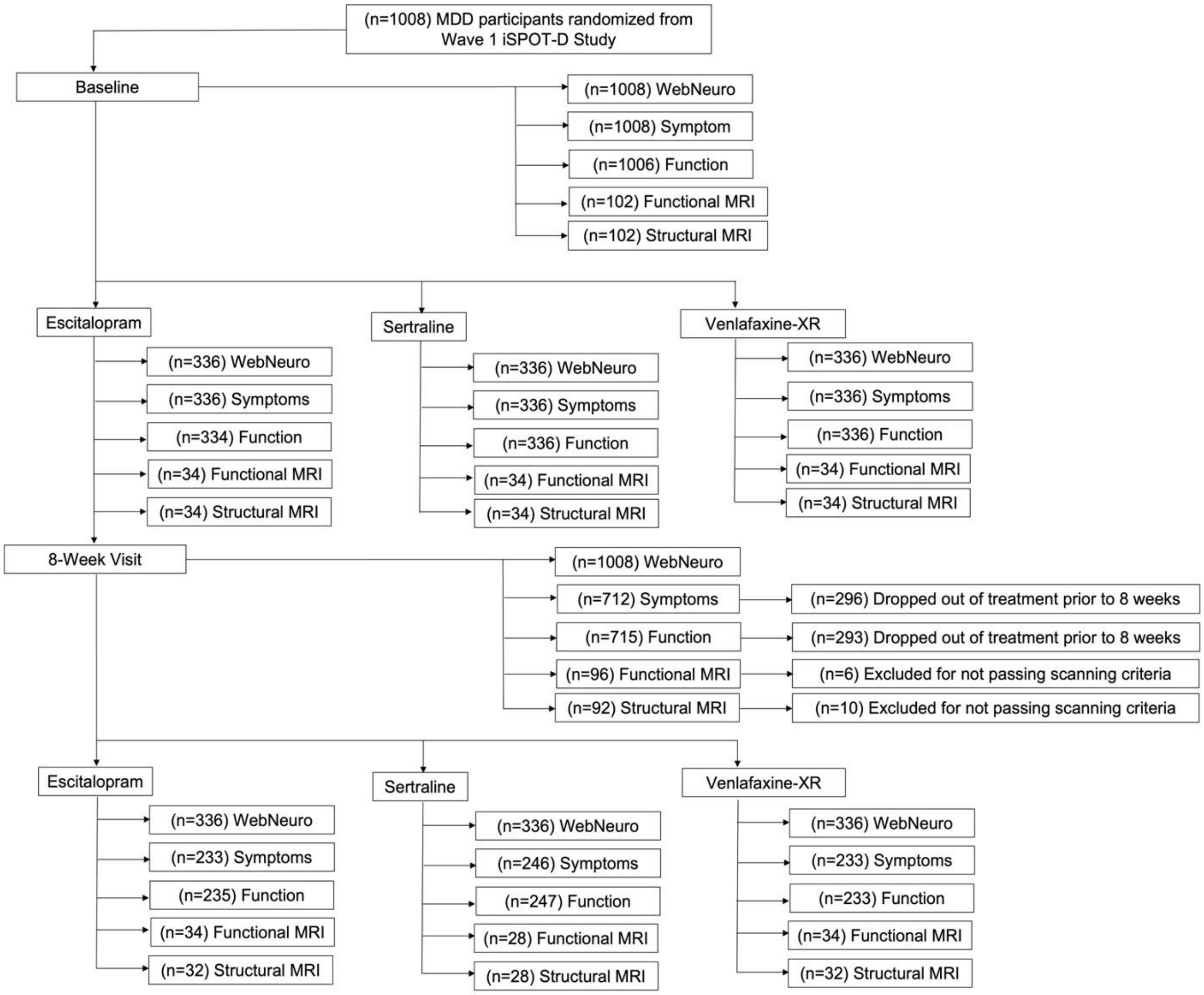

© 2023 Hack LM et al. *JAMA Network Open.*

eFigure 2. Scree Plot of the k-means Clustering Solutions with Different Numbers of Clusters

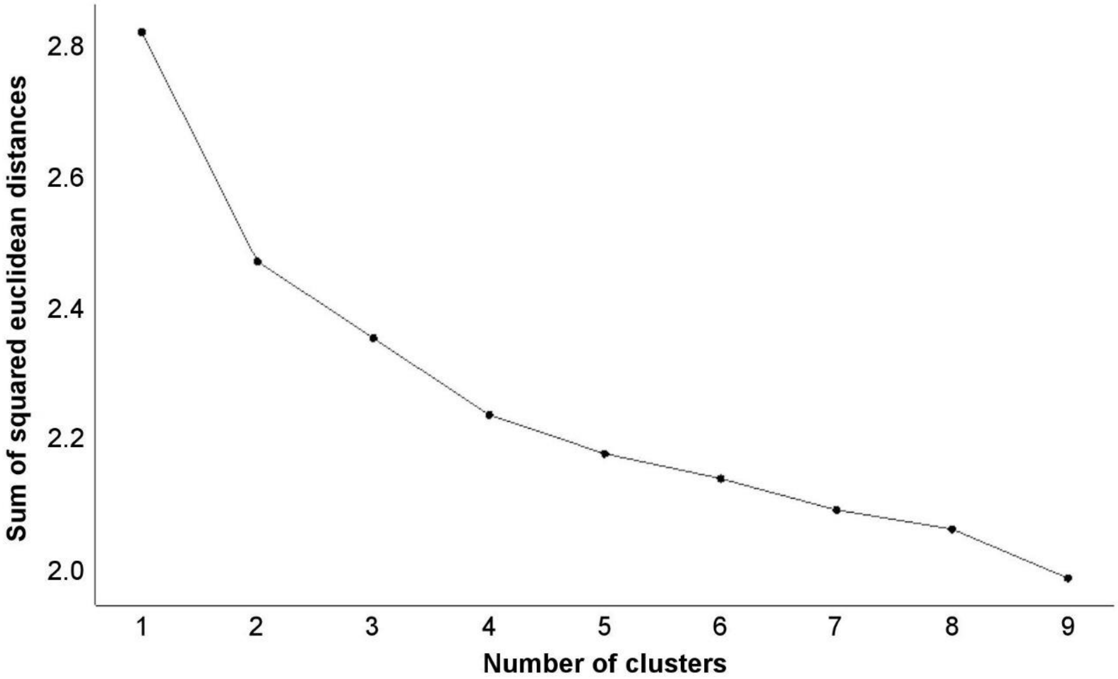

The scree plot indicates an elbow at 2 clusters, after which the line begins to flatten, indicating that additional clusters do not contribute to meaningfully to separating the data and suggesting k=2 as the best solution.

© 2023 Hack LM et al. *JAMA Network Open.*

eFigure 3. Post-treatment Cognitive Performance for Composite Measures in Cognitive Biotypes

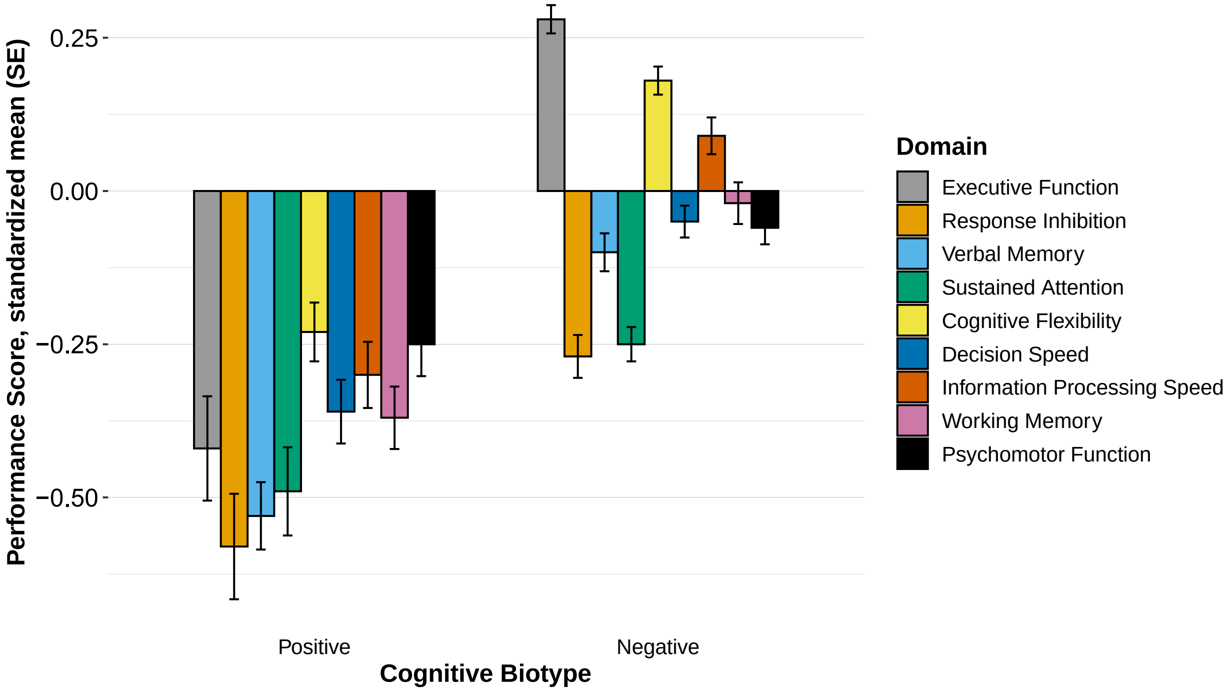

Standardized mean performance for post-treatment cognitive composite scores across 9 domains in the 2 cognitive biotypes. Error bars represent the standard error of the mean.

© 2023 Hack LM et al. *JAMA Network Open.*

eReferences

1. Sheehan DV, Lecrubier Y, Sheehan KH, et al. The Mini-International Neuropsychiatric Interview (M.I.N.I.): the development and validation of a structured diagnostic psychiatric interview for DSM-IV and ICD-10. *J Clin Psychiatry*. 1998;59 Suppl 20:22-33;quiz 34-57.
2. Hamilton M. A rating scale for depression. *J Neurol Neurosurg Psychiatry*. Feb 1960;23:56-62.
3. Clark CR, Paul RH, Williams LM, et al. Standardized assessment of cognitive functioning during development and aging using an automated touchscreen battery. *Arch Clin Neuropsychol*. Aug 2006;21(5):449-67. doi:10.1016/j.acn.2006.06.005
4. Baddeley A, Emslie H, Nimmo-Smith I. The Spot-the-Word test: a robust estimate of verbal intelligence based on lexical decision. *Br J Clin Psychol*. Feb 1993;32(1):55-65. doi:10.1111/j.2044-8260.1993.tb01027.x
5. Williams LM, Rush AJ, Koslow SH, et al. International Study to Predict Optimized Treatment for Depression (iSPOT-D), a randomized clinical trial: rationale and protocol. *Trials*. Jan 5 2011;12:4. doi:10.1186/1745-6215-12-4
6. Rowe DL, Cooper NJ, Liddell BJ, Clark CR, Gordon E, Williams LM. Brain structure and function correlates of general and social cognition. *J Integr Neurosci*. Mar 2007;6(1):35-74. doi:10.1142/s021963520700143x
7. Mathersul D, Palmer DM, Gur RC, et al. Explicit identification and implicit recognition of facial emotions: II. Core domains and relationships with general cognition. *J Clin Exp Neuropsychol*. Apr 2009;31(3):278-91. doi:10.1080/13803390802043619
8. Goldstein-Piekarski AN, Ball TM, Samara Z, et al. Mapping Neural Circuit Biotypes to Symptoms and Behavioral Dimensions of Depression and Anxiety. *Biol Psychiatry*. Mar 15 2022;91(6):561-571. doi:10.1016/j.biopsych.2021.06.024
9. Goldman HH, Skodol AE, Lave TR. Revising axis V for DSM-IV: a review of measures of social functioning. *Am J Psychiatry*. Sep 1992;149(9):1148-56. doi:10.1176/ajp.149.9.1148
10. Development of the World Health Organization WHOQOL-BREF quality of life assessment. The WHOQOL Group. *Psychol Med*. May 1998;28(3):551-8. doi:10.1017/s0033291798006667
11. Smith SM, Jenkinson M, Woolrich MW, et al. Advances in functional and structural MR image analysis and implementation as FSL. *Neuroimage*. 2004;23 Suppl 1:S208-19. doi:10.1016/j.neuroimage.2004.07.051
12. Korgaonkar MS, Grieve SM, Etkin A, et al. Magnetic Resonance Imaging of Major Depressive Disorder (MDD): First Planned Outcomes from the ISPOT-D Study. *Biological Psychiatry*. Apr 15 2012;71(8):210S-210S.
13. Williams LM, Korgaonkar, M.S. Song, Y.C., Paton, R. Eagles, S. Goldstein-Piekarski, A., Grieve, Stuart S.M., Harris, A.W.F., Usherwood, T., Etkin, A. Amygdala Reactivity to Emotional Faces in the Prediction of General and Medication-Specific Responses to Antidepressant Treatment in the Randomized iSPOT-D Trial. *Neuropsychopharmacology*. SEP 2015 2015;40(10):2398-2408. doi:10.1038/npp.2015.89
14. Ashburner J, Friston KJ. Unified segmentation. *Neuroimage*. Jul 01 2005;26(3):839-51. doi:10.1016/j.neuroimage.2005.02.018
15. Yarkoni T, Poldrack RA, Nichols TE, Van Essen DC, Wager TD. Large-scale automated synthesis of human functional neuroimaging data. *Nat Methods*. Jun 2011;8(8):665-70. doi:10.1038/nmeth.1635

© 2023 Hack LM et al. *JAMA Network Open.*

16. Williams LM. Precision psychiatry: a neural circuit taxonomy for depression and anxiety. *Lancet Psychiatry*. May 2016;3(5):472-80. doi:10.1016/S2215-0366(15)00579-9
17. Preacher KJ, Hayes AF. SPSS and SAS procedures for estimating indirect effects in simple mediation models. *Behav Res Methods Instrum Comput*. Nov 2004;36(4):717-31. doi:10.3758/bf03206553
18. Preacher KJ, Hayes AF. Asymptotic and resampling strategies for assessing and comparing indirect effects in multiple mediator models. *Behav Res Methods*. Aug 2008;40(3):879-91. doi:10.3758/brm.40.3.879
19. Dinga R, Schmaal L, Penninx BWJH, et al. Evaluating the evidence for biotypes of depression: Methodological replication and extension of. *Neuroimage Clin*. 2019;22:101796. doi:10.1016/j.nicl.2019.101796
20. McAllister TW, McDonald BC, Flashman LA, et al. Alpha-2 adrenergic challenge with guanfacine one month after mild traumatic brain injury: altered working memory and BOLD response. *Int J Psychophysiol*. Oct 2011;82(1):107-14. doi:10.1016/j.ijpsycho.2011.06.022
21. Goodkind MS, Gallagher-Thompson D, Thompson LW, et al. The impact of executive function on response to cognitive behavioral therapy in late-life depression. *Int J Geriatr Psychiatry*. Apr 2016;31(4):334-9. doi:10.1002/gps.4325

© 2023 Hack LM et al. *JAMA Network Open.*
