# Supplementary material for: A Cognitive Biotype of Depression and Symptoms, Behavior Measures, Neural Circuits, and Differential Treatment Outcomes: A Prespecified Secondary Analysis of a Randomized Clinical Trial
Source: JAMA Netw Open. 2023 Jun 15;6(6):e2318411. doi: 10.1001/jamanetworkopen.2023.18411 (PMC10273022; doi:10.1001/jamanetworkopen.2023.18411)
Supplement: Supplement 3. — Data Sharing Statement [file jamanetwopen-e2318411-s003.pdf]

Data Sharing Statement

Hack. A Cognitive Biotype of Depression and Symptoms, Behavior Measures, Neural Circuits, and Differential Treatment Outcomes. *JAMA Netw Open*. Published June 15, 2023. doi:10.1001/jamanetworkopen.2023.18411

Data
Data available: Yes
Data types: Deidentified participant data
How to access data: Data collected for the study, including individual participant data and a data dictionary defining each field in the set, will be made available after approval of a proposal with investigator support.
When available: With publication

Supporting Documents
Document types: None

Additional Information
Who can access the data: researchers whose proposed use of the data has been approved
Types of analyses: for a specified approved purpose
Mechanisms of data availability: after approval of a proposal
